# Gelsolin: A comprehensive pan-cancer analysis of potential prognosis, diagnostic, and immune biomarkers

**DOI:** 10.3389/fgene.2023.1093163

**Published:** 2023-03-23

**Authors:** Yiyang Wang, Xiaojuan Bi, Zhiwen Luo, Haiyan Wang, Dilimulati Ismtula, Chenming Guo

**Affiliations:** ^1^ Department of Breast Surgery, Center of Digestive and Vascular, The First Affiliated Hospital of Xinjiang Medical University, Urumqi, China; ^2^ State Key Laboratory of Pathogenesis, Prevention and Treatment of High Incidence Diseases in Central Asia, Clinical Medicine Institute, Urumqi, China

**Keywords:** pan-cancer, GSN, prognosis, serodiagnostics, tumor immunity, methylation

## Abstract

**Introduction:** Gelsolin (GSN), a calcium-regulated actin-binding protein, is out of balance in various cancers. It can mediate cytoskeletal remodeling and regulate epithelial-mesenchymal conversion (EMT), but the studies on GSN function in pan-cancer are limited.

**Methods:** We studied the transcription level, prognostic impact, diagnostic value, genetic, epigenetic modification, methylation level and immune significance of GSN in pan-cancer to fully comprehend the function of GSN in various malignancies based on multiple databases like The Cancer Genome Atlas (TCGA) and Gene Expression Omnibus (GEO).

**Results:** Pan-cancer research showed that GSN was downregulated in most tumors and expressed differently in immunological and molecular subtypes of many cancers. GSN had varying impacts on the prognosis of various tumor types. However, all had moderate to high diagnostic efficiency, and serum GSN had good diagnostic value in breast cancer patients (AUC = 0.947). Moreover, GSN was a distinguishing prognosis factor for some specific cancer types. The GSN protein was hypophosphorylated, and its promoter was hypermethylated in most cancers. GSN was linked to the infiltration level of several immunity cells and was essential in anti-tumor immune cell infiltration. KEGG and GSEA analyses showed that GSN was vital in the functions and proteoglycans processes in cancer, chemokine signaling pathway and other immune-related pathways, DNA methylation and cell cycle.

**Discussion:** In conclusion, GSN possesses the ability to be a predictive, diagnostic, and immune indicator in pan-cancer.

## 1 Introduction

The Gelsolin (GSN) gene found on chromosome 9q33.2 encodes a calcium-regulated actin regulatory protein of 782 amino acids. It comprises six gelsolin-like homologous domains (G1-G6). G1 and G4 bind two Ca^2+^ in two forms of shared Ca^2+^ with actin and completely wrapped Ca^2+^, while G2, G3, G5, and G6 each bind one Ca^2+^ in the form of wholly wrapped Ca^2+^, reorganizing the actin cytoskeleton, which affects cell motility, cell division and apoptosis (Choe et al., 2002; Nag et al., 2009). GSN is widely found in plasma and cytoplasm and acts as a transcriptional cofactor in signal transduction, and epigenetic changes affect its expression and activity and are essential for various diseases, including cancer, infection and inflammation, and heart damage (Li et al., 2012).

GSN is intimately linked to different types of tumor development as a vital controller of cell activity, division and death. The low GSN expression in colon cancer tissues is a favorable factor that improves the prognosis of colon cancer patients (Kim et al., 2018; Chen et al., 2019a) due to the silencing of GSN impedes colorectal cancer cell migration and invasiveness and induces cell cycle stagnation (Huang et al., 2022). Similarly, GSN, strongly expressed in bladder cancer tissues, is a major gene for poor prognosis. However, upregulated transcription factor 3 (ATF3) inhibits bladder cancer metastasis through upregulated GSN-mediated actin cytoskeletal remodeling (Yuan et al., 2013; Yang et al., 2020). In liver cancer, patients have a poor prognosis with high GSN expression, possibly because GSN overexpression increases the aggressiveness of cancer cells *via* controlling epithelial-mesenchymal transition (EMT) (Zhang et al., 2020b). Similarly, GSN, upregulated expression in non-small cell lung cancer (NSCLC) and breast cancer tissues, can mediate EMT action to increase cancer cell aggressiveness (Chen et al., 2015; Liu et al., 2021). Furthermore, upregulated GSN may suppress cancer cell proliferation and metastasis for glioblastoma and myelodysplastic syndrome (MDS) (Zhang et al., 2020a; Deng et al., 2020). For kidney cancer, knocking down GSN can inhibit cancer cell proliferation and metastasis (Xu et al., 2017). Accordingly, GSN may be an excellent prognostic biomarker in the above cancers, but research on its prognostic value in other cancers needs to be more extensive and clear.

GSN is divided into secretory and cytosolic types, and secretory GSN may have good value in diagnosing cancer. Serum GSN can act as a diagnostic marker for colon and esophageal adenocarcinoma (Shah et al., 2018; Chen et al., 2019b). Also, plasma GSN can be a factor to distinguish whether people with diabetes have pancreatic ductal adenocarcinoma (Peng et al., 2020). Additionally, the combined area under the curve (AUC) can reach 0.85, which is twice as accurate as the tumor marker CA19-9 alone (Peng et al., 2020). GSN is also associated with chemotherapy resistance, and GSN expression levels in gynecological and head and neck cancers tissues are positively correlated with *in vitro* and *in vivo* chemical resistance (Abedini et al., 2014; Wang et al., 2014). Since current research is limited to a single type of cancer, prognosis, or mechanism, investigating GSN function in a pan-cancer is crucial.

This study examined GSN expression and its diagnostic and predictive significance in pan-cancer and used the serum of breast cancer patients to verify it. Moreover, we explored protein phosphorylation, methylation modification, epigenetic alteration and other aspects and discussed the correlation between GSN expression and immunological response, immune cell infiltration and immune-related gene expression. Finally, functional and pathway enrichment analyses were carried out, providing ideas for further functional experiments. This pan-cancer analysis demonstrated the essential function of GSN in cancer.

## 2 Material and methods

### 2.1 Data downloading and GSN expression difference analysis

RNA-sequencing (RNA-seq) data and related medical data of the pan-cancer cohort (n = 15,776) were gained from UCSC XENA (https://xenabrowser.net/datapages/), containing genotype tissue expression (GTEx) of 33 different cancers and normal tissues from The Cancer Genome Atlas (TCGA). Transcripts Per Million (TPM) formatted expression spectrum data was transformed by log2 and merged with subsequent analyses. Data were validated using expression data from 8 datasets from the Gene Expression Omnibus (GEO) database, containing GSE42568 (platform: GPL570), GSE9750 (platform: GPL96), GSE20916 (platform: GPL570), GSE31547 (platform: GPL96), GSE54129 (platform: GPL570), GSE225638 (platform: GPL570), GSE43176 (platform: GPL96) and GSE15471 (platform: GPL570).

### 2.2 Explore diagnostic and prognostic potential of GSN

The Cox regression model examined the connection between GSN expression and outcome in patients with each tumor. Information was obtained regarding patient survival comprises overall survival (OS), disease-specific survival (DSS), disease-free survival (DFS), and progression-free survival (PFS). For conducting COX regression and plotting Kaplan-Meier (KM) curves, the “survival” and “survminer” packages were used. Forest and venn plots were created utilizing the “ggplot2” package to show the finding. The PrognoScan database (http://dna00.bio.kyutech.ac.jp/PrognoScan/index.html) analyzed 12 datasets involving 8 tumors to examine the connection between GSN expression and patient survival prognoses.

The R package “pROC” was utilized to conduct receiver operating characteristic (ROC) curve analysis to explore GSN predicted values in TCGA tumor tissues and the values in the matching GTEx and TCGA normal tissues. AUC between 0.7 and 0.9 indicates that TUBA1B has a specific diagnostic ability. AUC > 0.9 indicated good diagnostic ability.

### 2.3 Serum sample collection and ELISA

A total of 37 breast cancer patients who had surgery in the First Affiliated Hospital of Xinjiang Medical University were selected. Simultaneously, 31 healthy women who had a physical examination were randomly chosen as normal controls. Inclusion criteria were: ① All patients diagnosed with medical treatment for the first time, and blood samples were taken before systemic chemotherapy, radiotherapy, endocrine therapy, targeted therapy and surgical treatment. ② All patients pathologically diagnosed with primary breast invasive carcinoma (BRCA). ③ All patients had complete medical records. Exclusion criteria were: patients with other malignant tumors, autoimmune diseases, liver disease, kidney disease, and infectious diseases. All subjects signed informed consent, and the study complied with the Declaration of Helsinki of the World Medical Association. Also, the First Affiliated Hospital Ethics Committee of Xinjiang Medical University accepted this study (20220309–167). Venous blood was collected from the fasting elbows of all subjects into vacuum collection vessels without any anticoagulant, and the supernatant was collected after centrifugation for examination. ELISA detected serum GSN content (ab270215, Abcam, United Kingdom).

### 2.4 Nomograms development and calibration

First, we assessed risk factors that affected patient prognosis using univariate and multivariate Cox regressions; therefore, variables with *p*-values < 0.1 was employed for subsequent multivariate Cox analyses. GSN expression was split into high- and low-expression groups utilizing the average to be the threshold and then included as an independent factor. The criteria selected in the multivariate COX regression analysis were incorporated into the nomogram, and the consistency index (C-index) determined the predictive validity of nomogram, where 1,000 was used as a replicates number. Plot calibration curves were conducted to contrast the predicted and the real operating systems.

### 2.5 Use of online databases

The connection between GSN expression and different human cancer subtypes was examined using the “Subtypes” module of TISIDB database (http://cis.hku.hk/TISIDB/index.php) obtaining the connection between methylation levels and the degree of immune cell infiltration (Ru et al., 2019).

The “TCGA” module of UALCAN database (http://ualcan.path.uab.edu/index.html) was employed to contrast the GSN promoter methylation levels of various malignancies between normal and TCGA samples (Chandrashekar et al., 2022). From the “CPTAC” module, the protein content and phosphorylation level of pan-cancer tissue and its corresponding normal tissue were analyzed.

To verify the differential expression of GSN at the protein level, immunohistochemical images of nine cancer tissues and the matching healthy tissues with various GSN expressions and protein content were gained from the Human Protein Atlas (HPA) database (https://www.proteinatlas.org/) (Thul and Lindskog, 2018).

The “Mutation” module in the Gene Set Cancer Analysis (GSCA) database (http://bioinfo.life.hust.edu.cn/GSCA/#/) was employed to assess the copy number variation% (CNV%) within every cancer, the connection between the expression of GSN and its methylation levels and CNV shifts, and the impacts of GSN methylation levels and CNV changes on pan-cancer prognosis (Liu et al., 2018).

The “OncoPrint” module of cBioPortal database (https://www.cbioportal.org) (Gao et al., 2013) was employed to examine the levels of GSN genetic changes in the “TCGA Pan-Cancer Atlas Studies” dataset (10.443 samples with mutation data in 32 studies). The “Cancer Types Summary” module assesses the recurrence of GSN changes, genetic mutation number, mutation type, and CNV in every cancer form. The GSN mutation site was evaluated by the “mutation” module and demonstrated in the 3D structure of its protein.

The GSN percentage within each CNV and Single Nucleotide Variation (SNV) type in pan-cancer, was obtained from The Catalogue of Somatic Mutations in Cancer (COSMIC) (https://cancer.sanger.ac.uk/cosmic).

### 2.6 Association of GSN with tumor immunity

First, we examined the associations between GSN and tumor mutational load (TMB) and microsatellite instability (MSI) in several cancers using Sangerbox 3.0 (http://vip.sangerbox.com/) online database. The “GSVA” and “org.Hs.eg.db” tools were performed to compute StromalScore, ImmuneScore, and ESTIMATEScore in the ESTIMATE algorithm (Yoshihara et al., 2013). Eight genes were selected as immune checkpoint-associated transcripts, and the connection between their expression and GSN expression in pan-cancer was evaluated. Moreover, a list of genes for immune activator, immunosuppressive, chemokine, chemokine receptor, and major histocompatibility complex (MHC) molecules was gained from the Gene Set Enrichment Analysis (GSEA) database (https://www.gsea-msigdb.org/gsea/msigdb/index.jsp). The association between GSN expression levels and immune-related gene expression was examined utilizing the Spearman correlation coefficient.

The single-sample GSEA (ssGSEA) algorithm was employed to assess the invasion level of 24 immunological cells in pan-cancer (Bindea et al., 2013). The EPIC, TIMER, CIBERSORT, MCPCOUNTER algorithm of the “Immune” module of TIMER2.0 database (http://timer.cistrome.org/) was utilized to study the link between GSN expression and levels of immune cell infiltration in pan-cancer, containing cancer-associated fibroblasts (CAFs), CD8+ T cells, CD4+ T cells, regulatory T cells (Tregs), B cells, neutrophils, monocytes, myeloid dendritic cells (mDCs), macrophages, and natural killer cells (NKs) (Li et al., 2020). The “Gene_Corr” module was utilized to examine the association between GSN expression and biomarker expression of CAFs and mDCs.

### 2.7 Function and pathway enrichment analysis

GSN-targeted binding proteins were studied utilizing the STRING database (https://string-db.org/). Experimentally detected GSN-binding proteins were created using setting STRING parameters, and protein-protein interaction (PPI) networks were formed. The “Similar Genes Detection” module in GEPIA2 (http://gepia2.cancer-pku.cn/#index) was utilized to create the first 100 genes co-expressed with GSN (Tang et al., 2019). The “clusterProfiler” and the “org.Hs.eg.db” tools’ were utilized to perform enrichment analysis of the GSN functionality, and a bubble chart shows five of each item. Moreover, GSEA was used to elucidate the functional pathways of differential GSN in the two expression groups of varying cancer cohorts, with a gene set of “c2.cp.v7.2.symbols.gmt” from MSigDB, and all analyses were repeated 5,000 times. A ridge plot shows the highest 15 “Reactom pathways” for each cancer type.

### 2.8 Statistical analysis

R software (vs. 4.0.3, https://www. R-project.org/) for statistical analysis, the “ggplot2” package for a visualization, was used for statistical analyses. The Mann-Whitney U test was utilized to examine variations in expression levels of GSN in unmatched samples, and Wilcoxon signed rank test was employed for paired samples. The Spearman correlation coefficient was utilized to study the association between GSN expression and m6A methylation regulators, TMB, MSI, immune score, and immune-related genes. *p* < 0.05 was regarded as statistically significant.

## 3 Results

### 3.1 Differences of GSN expression in pan-carcinoma and its subtypes

**FIGURE 1:**
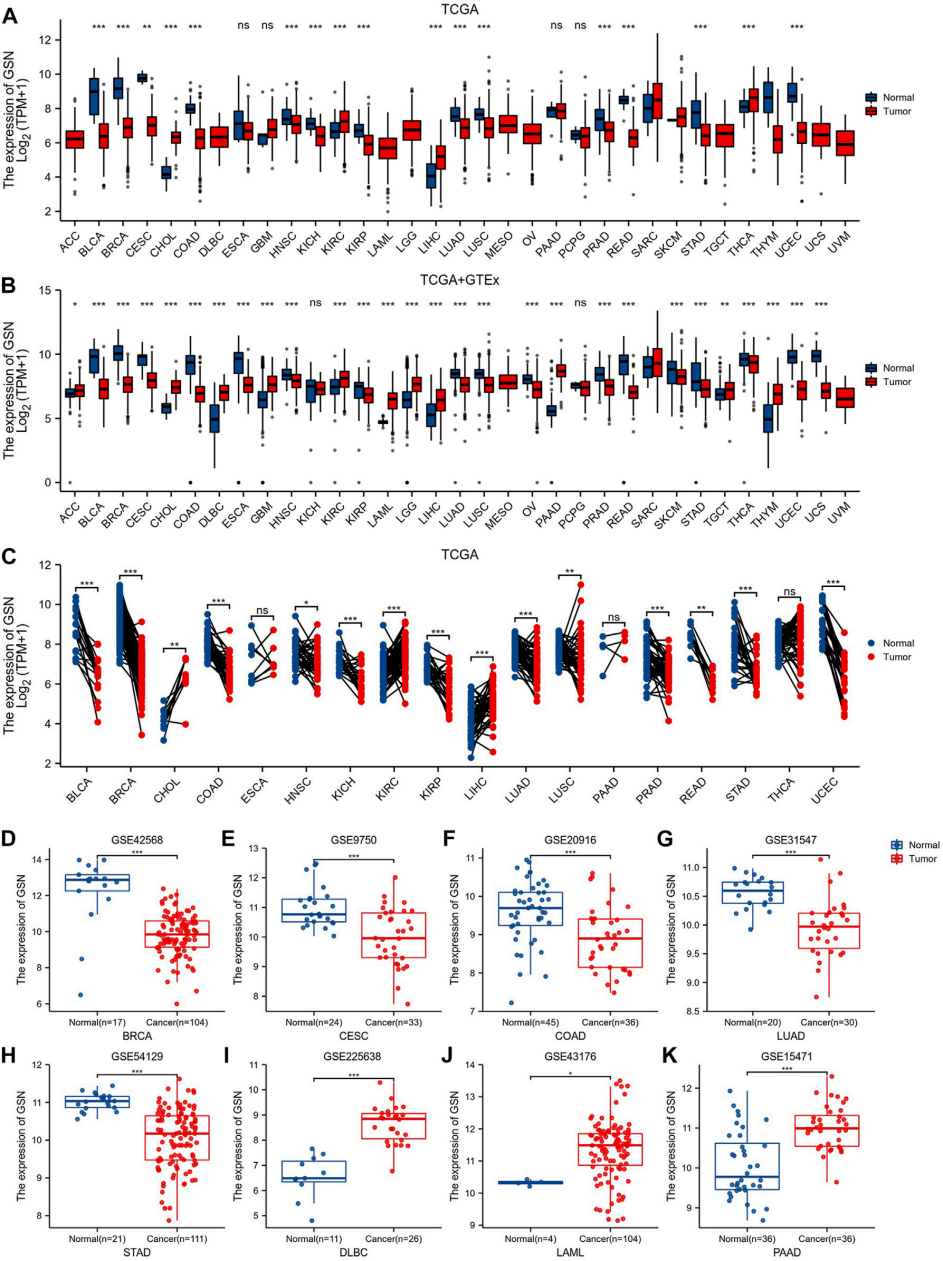
Differences in GSN expression in 33 cancers. **(A)** GSN mRNA expression difference between TCGA tumor and normal tissues. **(B)** GSN mRNA expression difference between tumor and normal tissues with data from the TCGA and GTEx. **(C)** GSN mRNA expression in TCGA tumor and paired normal tissues. The differential expression of GSN was analyzed using BRCA (GSE42568) **(D)**, CESC (GSE9750) **(E)**, COAD (GSE20916) **(F)**, LUAD (GSE31547) **(G)**, STAD (GSE54129) **(H)**, DLBC (GSE225638) **(I)**, LAML (GSE43176) **(J)**, and PAAD (GSE15471) **(K)** datasets in GEO databases. (*p < 0.05, **p < 0.01, ***p < 0.001).

Figure 1A shows GSN expression levels in bladder urothelial carcinoma (BLCA), BRCA, cervical squamous cell carcinoma and endocervical adenocarcinoma (CESC), colon adenocarcinoma (COAD), head and neck squamous cell carcinoma (HNSC), kidney chromophobe (KICH), kidney renal papillary cell carcinoma (KIRP), lung adenocarcinoma (LUAD), lung squamous cell carcinoma (LUSC), prostate adenocarcinoma (PRAD), rectum adenocarcinoma (READ), stomach adenocarcinoma (STAD), and uterine corpus endometrial carcinoma (UCEC) was reduced contrasted with that of healthy tissues. In contrast, cholangiocarcinoma (CHOL), kidney renal clear cell carcinoma (KIRC), liver hepatocellular carcinoma (LIHC) and thyroid carcinoma (THCA) GSN mRNA expression patterns were elevated compared with the matching healthy tissue levels.

GTEx normal tissue was matched to TCGA cancer tissue to create more persuasive outcomes. Furthermore, we found a significant elevation in GSN expression of 11 malignancies: adrenocortical carcinoma (ACC), CHOL, lymphoid neoplasm diffuse large B-cell lymphoma (DLBC), glioblastoma multiforme (GBM), KIRC, acute myeloid leukemia (LAML), brain lower grade glioma (LGG), LIHC, pancreatic adenocarcinoma (PAAD), testicular germ cell tumors (TGCT), thymoma (THYM). Conversely, in 17 malignancies: BLCA, BRCA, CESC, COAD, esophageal carcinoma (ESCA), HNSC, KIRP, LUAD, LUSC, ovarian serous cystadenocarcinoma (OV), PRAD, READ, skin cutaneous melanoma (SKCM), STAD, THCA, UCEC and uterine carcinosarcoma (UCS), GSN expression was downregulated contrasted with in healthy tissue (*p* < 0.05; Figure 1B). In both data, we found different results at GSN expression levels in THCA. In paired samples from 18 malignancies, we discovered that GSN mRNA expression patterns were elevated significantly in malignancies such as CHOL, KIRC and LIHC compared to neighboring normal tissues and significantly downregulated in cancers: BLCA, BRCA, COAD, HNSC, KICH, KIRP, LUAD, LUSC, PRAD, READ, STAD and UCEC (*p* < 0.05; Figure 1C). By analyzing the GEO dataset, we discovered that GSN expression patterns were reduced significantly in BRCA (*p* = 6.2e-07), CESC (*p* = 1.8e-04), COAD (*p* = 2.9e-04), LUAD (*p* = 9.2e-06) and STAD (*p* = 4.4e-09) compared to the corresponding normal tissues (Figures 1D–H). Concurrently, DLBC (*p* = 9.7e-09), LAML (*p* = 0.01) and PAAD (*p* = 3.5e-07) were significantly elevated (Figures 1I–K).

**FIGURE 2:**
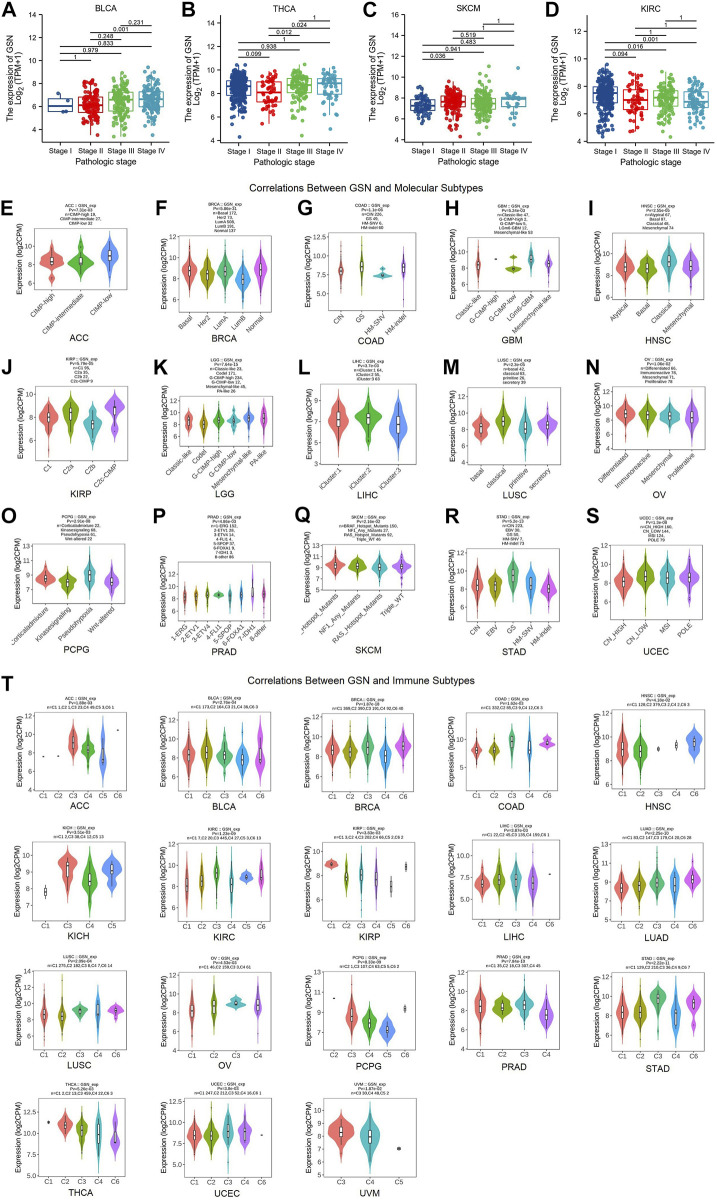
Correlation of GSN expression with different tumor stages, molecular subtypes, and immune subtypes. Correlation between GSN expression and different tumor stages, including BLCA **(A)**, THCA **(B)**, SKCM **(C)**, KIRC **(D)**. Correlations between molecular subtypes and GSN expression across TCGA tumors, including **(E)** ACC; **(F)** BRCA; **(G)** COAD; **(H)** GBM; **(I)** HNSC; **(J)** KIRP; **(K)** LGG; **(L)** LIHC; **(M)** LUSC; **(N)** OV; **(O)** PCPG; **(P)** PRAD; **(Q)** SKCM; **(R)** STAD; **(S)** UCEC. **(T)** Correlations between immune subtypes and GSN expression across TCGA tumors, including ACC, BLCA, BRCA, COAD, HNSC, KICH, KIRC, KIRP, LIHC, LUAD, LUSC, OV, PCPG, PRAD, STAD, THCA, UCEC and UVM. C1 (wound healing), C2 (IFN-g dominant), C3 (inflammatory), C4 (lymphocyte deplete), C5 (immunologically quiet), and C6 (TGF-b dominant).

Herein, the correlation between GSN expression in different tumor stages was found that in tumors with decreased GSN expression, including BLCA, THCA and SKCM, the decrease in GSN expression was more significant in early cancers (Figures 2A–C). In tumors with elevated GSN expression, including KIRC, the increase in GSN expression was more significant in early cancers (Figure 2D). This suggested that GSN has the potential to serve as an essential clinical indicator for the early diagnosis of malignancy in these cancers.

Then, we employed the TISIDB database to investigate the differential expression of GSN in various pan-cancer immunological as well as molecular subtypes. Figures 2E–S show that GSN expression varied in 15 cancer subtypes with distinct molecular subtypes. For tumor types with high GSN expression, the molecular subtype of CIMP-low in ACC exhibited the most significant GSN expression (Figure 2E), LGm6-GBM for GBM (Figure 2H), Mesenchymal-like for LGG (Figure 2K) and iCluster:2 for LIHC (Figure 2L). Meanwhile, for tumor types with low GSN expression, GSN expression was the lowest in the molecular subtype of LumB for BRCA (Figure 2F), HM-SNV for COAD (Figure 2G), Basal for HNSC (Figure 2I), C2b for KIRP (Figure 2J), primitive for LUSC (Figure 2M), Proliferative for OV (Figure 2N), Wnt-altered for PCPG (Figure 2O), 1-ERG for PRAD (Figure 2P), RAS_Hotspot_Mutants for SKCM (Figure 2Q), HM-indel for STAD (Figure 2R) as well as CN_HIGH for UCEC (Figure 2S).

Moreover, we discovered that GSN expression was significantly associated with various immunological subtypes of 18 malignancies: ACC, BLCA, BRCA, COAD, HNSC, KICH, KIRC, KIRP, LIHC, LUAD, LUSC, OV, PCPG, PRAD, STAD, THCA, UCEC and UVM. In many cancers, GSN expression was highest in the C3 (inflammatory) immune subtype and lowest in the C4 (lymphocyte-depleted) immune subtype (Figure 2T). In summary, immune and molecular subtypes exhibited various GSN expressions.

### 3.2 Prognostic and diagnostic value of GSN in pan-carcinoma

**FIGURE 3:**
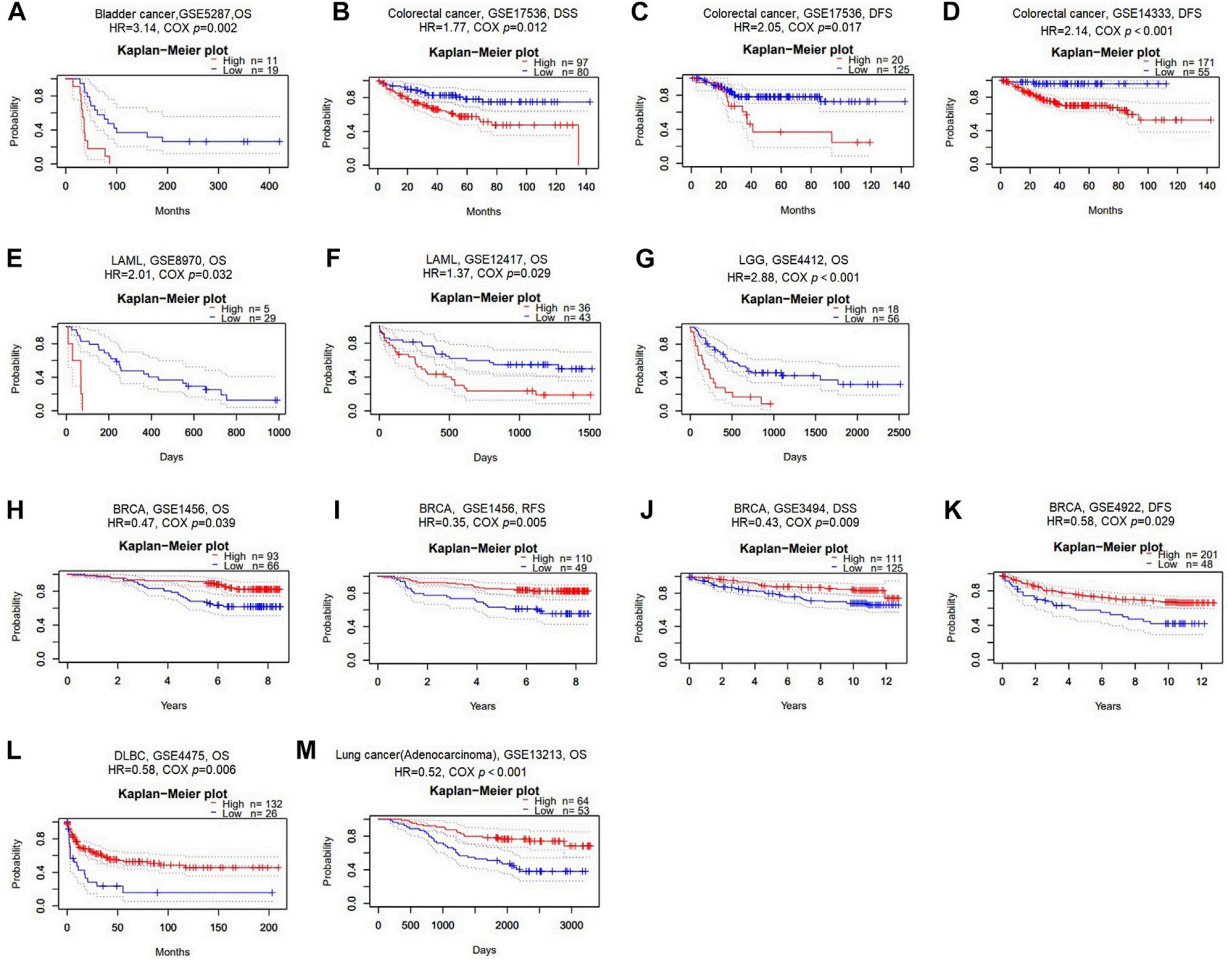
Survival analysis of GSN across different cancer types in the GEO and TCGA datasets. Kaplan-Meier plots of GSN in eleven datasets including GSE5287, bladder cancer, OS **(A)**; GSE17536, colorectal cancer, DSS and DFS **(B–C)**; GSE14333, colorectal cancer, DFS **(D)**; GSE8970, LAML, OS **(E)**; GSE12417, LAML, OS **(F)**; GSE4412, LGG, OS **(G)**; GSE1456, BRCA, OS and RFS **(H–I)**; GSE3494, BRCA, DSS **(J)**; GSE4922, BRCA, DFS **(K)**; GSE4475, DLBC, OS **(L)**; GSE13213, LUAD, OS **(M)**.

To comprehend if GSN expression influences the outcome of cancer patients, we carried out a survival analysis according to GSN expression in cancer patients using the PrognoScan database. Herein, 11 datasets were included: (GSE5287, GSE17536, GSE14333, GSE8970, GSE12417, GSE4412, GSE1456, GSE3494, GSE4922, GSE4475, and GSE13213) from bladder cancer, colorectal cancer, LAML, LGG, BRCA, DLBC, and LUAD. Higher GSN expression was related to worse outcomes in bladder cancer patients, colorectal cancer, LAML, and LGG (Cox *p* < 0.05; Figures 3A–G). Lower GSN expression was related to poorer prognoses in BRCA, DLBC, and LUAD patients (Cox *p* < 0.05; Figures 3H–M).

**FIGURE 4:**
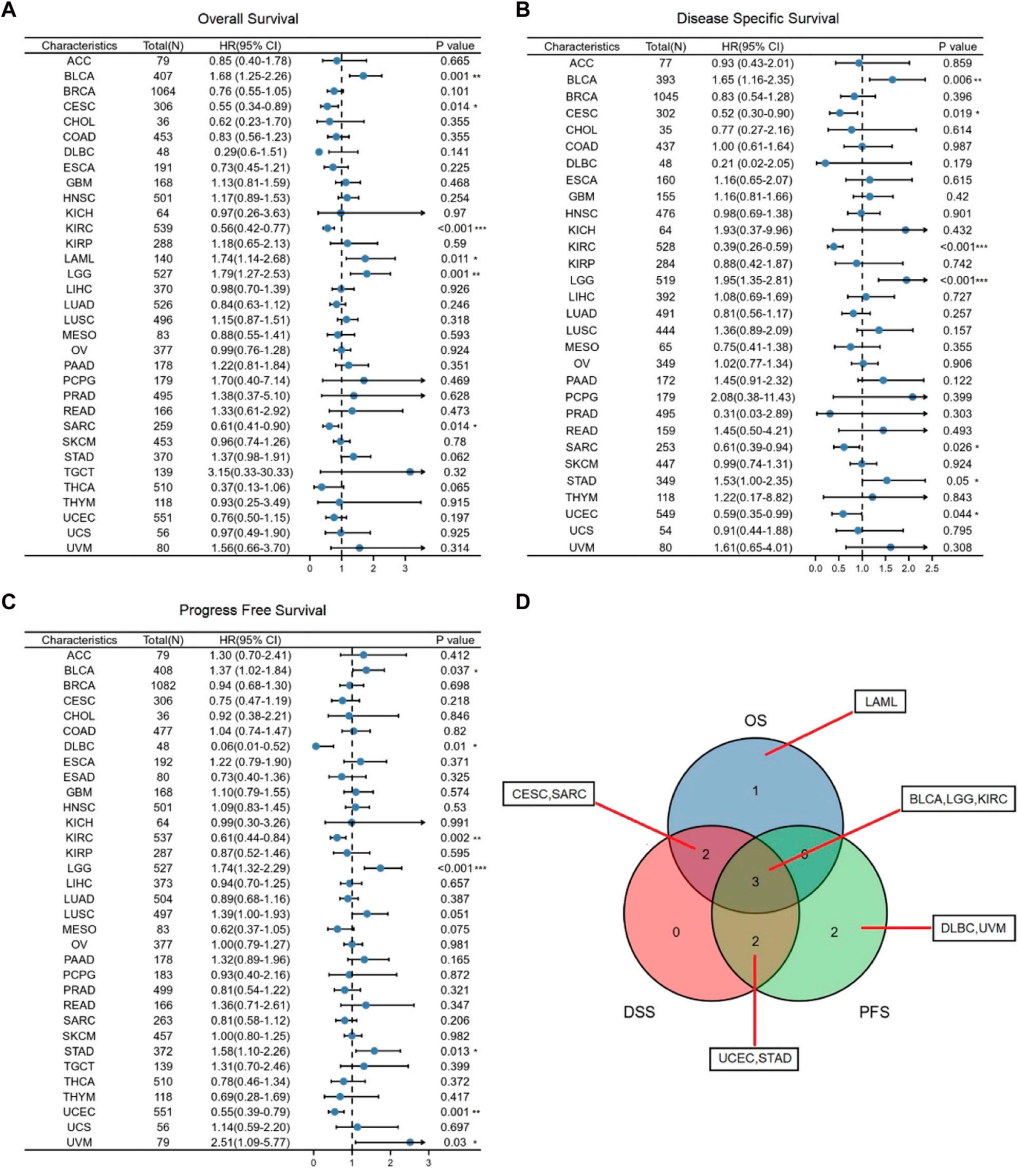
Association between GSN expression and prognosis in cancer patients. **(A)** Association between GSN expression and OS in cancer patients. **(B)** Association between GSN expression and DSS in cancer patients. **(C)** Association between GSN expression and PFS in cancer patients. **(D)** The venn diagram shows the intersection of OS, DS, PFS for different cancers. (*p < 0.05, **p < 0.01, ***p < 0.001).

Next, we utilized TCGA RNA-seq data to examine the prognostic value of GSN, including OS, DSS, and PFS. For OS, we found that low-expression GSN was an adverse factor affecting OS in patients with CESC, KIRC, SARC, and low-expression GSN was a protective variable for BLCA, LAML and LGG patients (*p* < 0.05; Figure 4A and Supplementary Figure S1A). For DSS, low GSN expression was a negative factor affecting DSS in patients with CESC, KIRC, SARC, and UCEC, while it is a preventative variable for BLCA, LGG, and STAD patients (*p* < 0.05; Figure 4B and Supplementary Figure S1B). Similarly, low-expression GSN was a detrimental factor affecting PFS in patients with DLBC, KIRC, and UCEC. In contrast, reduced GSN expression was a protective factor affecting PFS in BLCA, LGG, STAD, and UVM patients (*p* < 0.05; Figure 4C and Supplementary Figure S1C). The Venn plot shows that GSN affects three prognoses (OS, DSS, PFS) for patients with BLCA, LGG, and KIRC, revealing that GSN can be a crucial variable in the outcome of such cancers (Figure 4D).

**FIGURE 5:**
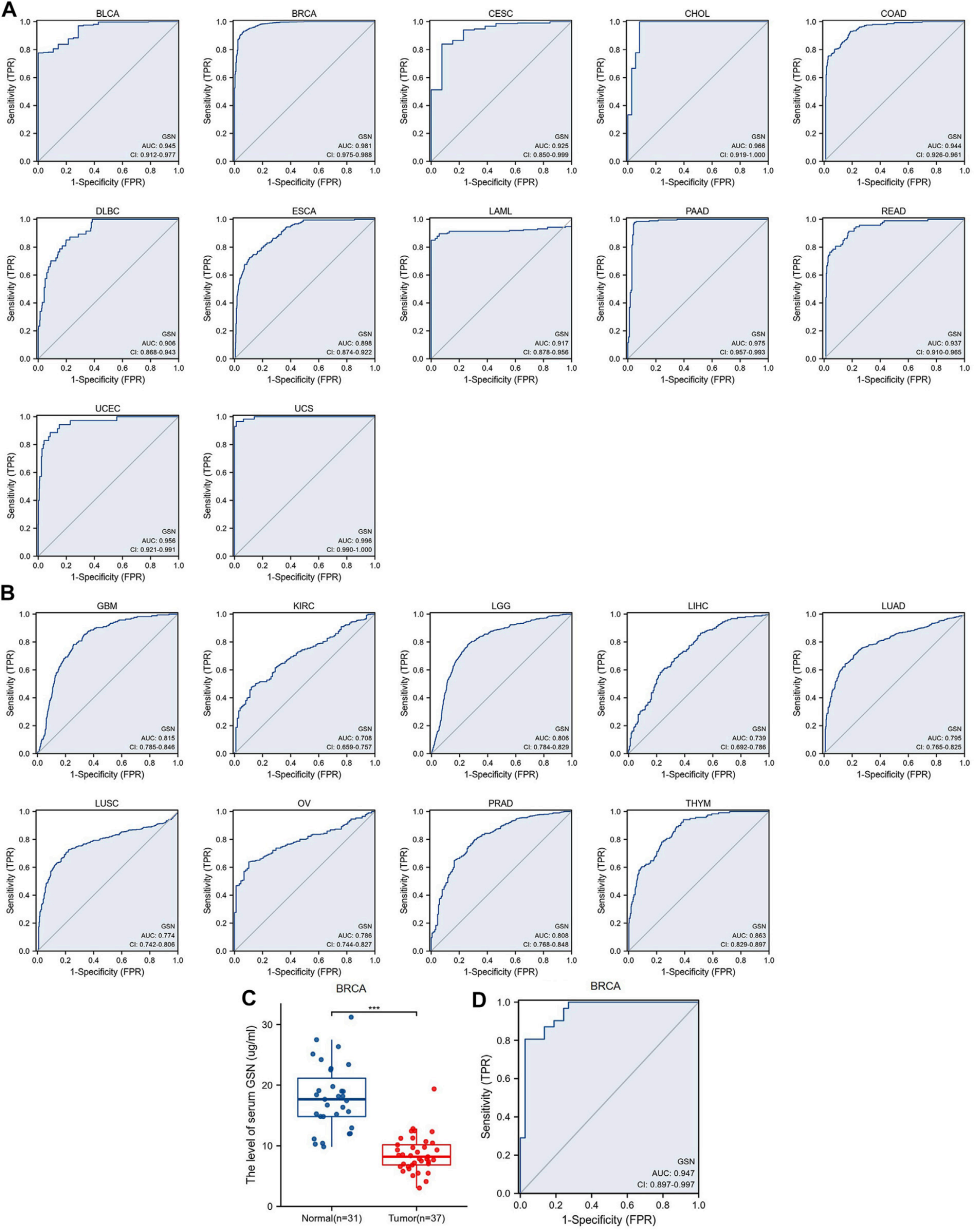
Receiver operating characteristic (ROC) curve of GSN expression in pan-carcinoma and determination of serum GSN in breast cancer patients. **(A)** GSN expresses cancers of good diagnostic value (AUC>0.9), including BLCA, BRCA, CESC, CHOL, COAD, DLBC, ESCA, LAML, PAAD, READ, UCEC, UCS. **(B)** GSN expresses cancer with some diagnostic value (AUC>0.7), including GBM, KIRC, LGG, LIHC, LUAD, LUSC, OV, PRAD, THYM. **(C)** GSN protein content in serum of breast cancer patients. **(D)** Diagnostic ROC curve of serum GSN for breast cancer. (***p < 0.001).

We introduced ROC curve analysis to investigate the possible diagnosis of GSN in pan-cancer. Findings showed that GSN had good diagnostic capabilities (AUC > 0.9), including BLCA (0.945), BRCA (0.981), CESC (0.925), CHOL (0.966), COAD (0.944), DLBC (0.906), ESCA (0.898), LAML (0.917), PAAD (0.975), READ (0.937), UCEC (0.956) and UCS (0.996) (Figure 5A). GSN showed some diagnostic potential (AUC > 0.7) in some tumors, including GBM (0.815), KIRC (0.708), LGG (0.806), LIHC (0.739), LUAD (0.795), LUSC (0.774), OV (0.786), PRAD (0.808) and THYM (0.863) (Figure 5B). GSN showed the highest predictive significance for breast cancer patients by excluding cancers with small sample sizes. Therefore, we collected serum from 37 breast cancer patients and 31 normal people to verify the diagnostic potential of serum GSN for BRCA. Compared to normal people (8.603 ± 3.007 μg/mL), the serum GSN level of breast cancer patients (17.970 ± 5.406 μg/mL) was significantly reduced (*p* < 0.001; Figure 5C). Next, we used ROC curve analysis to examine if serum GSN possesses diagnostic significance of BRCA. The findings revealed that the AUC of serum GSN was 0.947 (cut-off value: 12.883; sensitivity: 97.3%; specificity: 80.0%; Figure 5D). The results were similar to the above ROC curve analysis data, revealing that serum GSN might be useful in diagnosing BRCA.

Overall, GSN had a modest to the robust ability to differentiate cancer and healthy tissue for most cancers. Serum GSN was validated to have an excellent ability to diagnose breast cancer patients.

### 3.3 GSN is an independent variable in prognosis of some malignancies

We performed univariate and multivariate regression analyses for eight cancer types to investigate the risk factors influencing OS in cancer patients. Herein, the univariate COX regression analysis incorporated cancer types with *p* < 0.1: BLCA, CESC, KIRC, LAML, LGG, SARC, STAD and THCA. For BLCA, multivariate analysis showed that the main treatment result (partial response (PR)/complete response (CR), hazard ratio (HR) = 0.352, *p* < 0.001) and GSN expression (high GSN, HR = 1.712, *p* = 0.043) were independent factors affecting patient OS (Table S1A). For CESC, T stage (T3/T4, HR = 10.091, *p* = 0.002), N stage (N1, HR = 2.722, *p* = 0.043), clinical stage (stage III, HR = 0.119, *p* = 0.034), and main treatment result (PR/CR, HR = 0.160, *p* < 0.001) were independent predictive variables (Supplementary Table S1B). For KIRC, the main treatment result (PR/CR, HR = 0.120, *p* = 0.002) was the only independent predictive variable (Supplementary Table S1C). For LAML, age (>60, HR = 2.751, *p* < 0.001), cytogenetic risk (intermediate, HR = 2.767, *p* = 0.005) (poor, HR = 2.893, *p* = 0.009), and GSN expression (high GSN, HR = 1.928, *p* = 0.004) were independent predictive variables (Supplementary Table S1D). For LGG, WHO grade (G3, HR = 2.871, *p* < 0.001), main treatment result (PR/CR, HR = 0.210, *p* < 0.001), age (>40, HR = 2.939, *p* < 0.001), and GSN expression (high GSN, HR = 1.793, *p* = 0.004) were independent prognostic factors (Supplementary Table S1E). For SARC, residual tumor (R1, HR = 2.192, *p* = 0.012) (R2, HR = 10.143, *p* < 0.001), metastasis (transferred group, HR = 2.738, *p* < 0.001), and GSN expression (high GSN, HR = 0.350, *p* < 0.001) were independent predictive variables (Supplementary Table S1F). For STAD, N stage (N3, HR = 2.899, *p* = 0.043), primary therapy outcome (PR/CR, HR = 0.295, *p* < 0.001), age (>65, HR = 1.676, *p* = 0.019), and GSN expression (high GSN, HR = 1.597, *p* = 0.033) were independent predictive variables (Supplementary Table S1G). For THCA, the pathologic stage (stage III/IV, HR = 9.573, *p* = 0.010) was the only independent predictive variable (Supplementary Table S1H).

**FIGURE 6:**
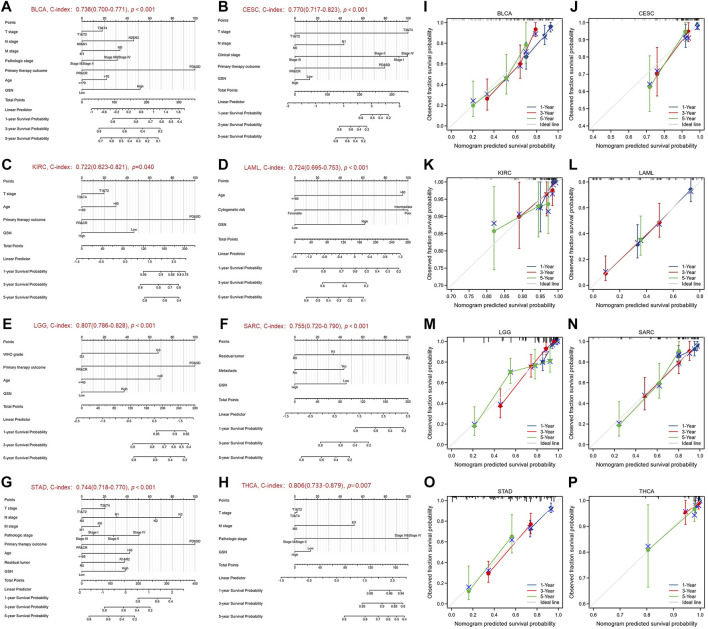
Nomograms and calibration curves predicting patient OS in 8 cancers. Nomograms of BLCA **(A)**; CESC **(B)**; KIRC **(C)**; LAML **(D)**; LGG **(E)**; SARC **(F)**; STAD **(G)**; THCA **(H)**. Calibration curves of BLCA **(I)**; CESC **(J)**; KIRC **(K)**; LAML **(L)**; LGG **(M)**; SARC **(N)**; STAD **(O)**; THCA **(P)**. The horizontal and vertical coordinates are the model predicted and actually observed survival probability, respectively. The closer each line is to the ideal line, the better the model.

We used the factors that had *p* < 0.1 in univariate COX regression analysis to construct predictive nomograms and calibrations. The findings revealed that the C-index of nomogram in BLCA was 0.736 (0.700–0.771, Figure 6A), in CESC, was 0.770 (0.717–0.823) (Figure 6B), in KIRC was 0.722 (0.623–0.821, Figure 6C), in LAML was 0.724 (0.695–0.753, Figure 6D), in LGG was 0.807 (0.786–0.828, Figure 6E), in SARC, was 0.755 (0.720–0.790, Figure 6F), in STAD was 0.744 (0.718–0.770, Figure 6G), in THCA, was 0.806 (0.733–0.879, Figure 6H). We then calibrated each nomogram to assess the reliability of this model. Except for THCA, the calibration curves for the remaining seven cancer types were close to the ideal line (Figures 6I–P). Therefore, GSN can be used to predict patient outcomes for these tumors independently.

### 3.4 Differences in protein content, phosphorylation and methylation modification levels of GSN in pan-cancer

**FIGURE 7:**
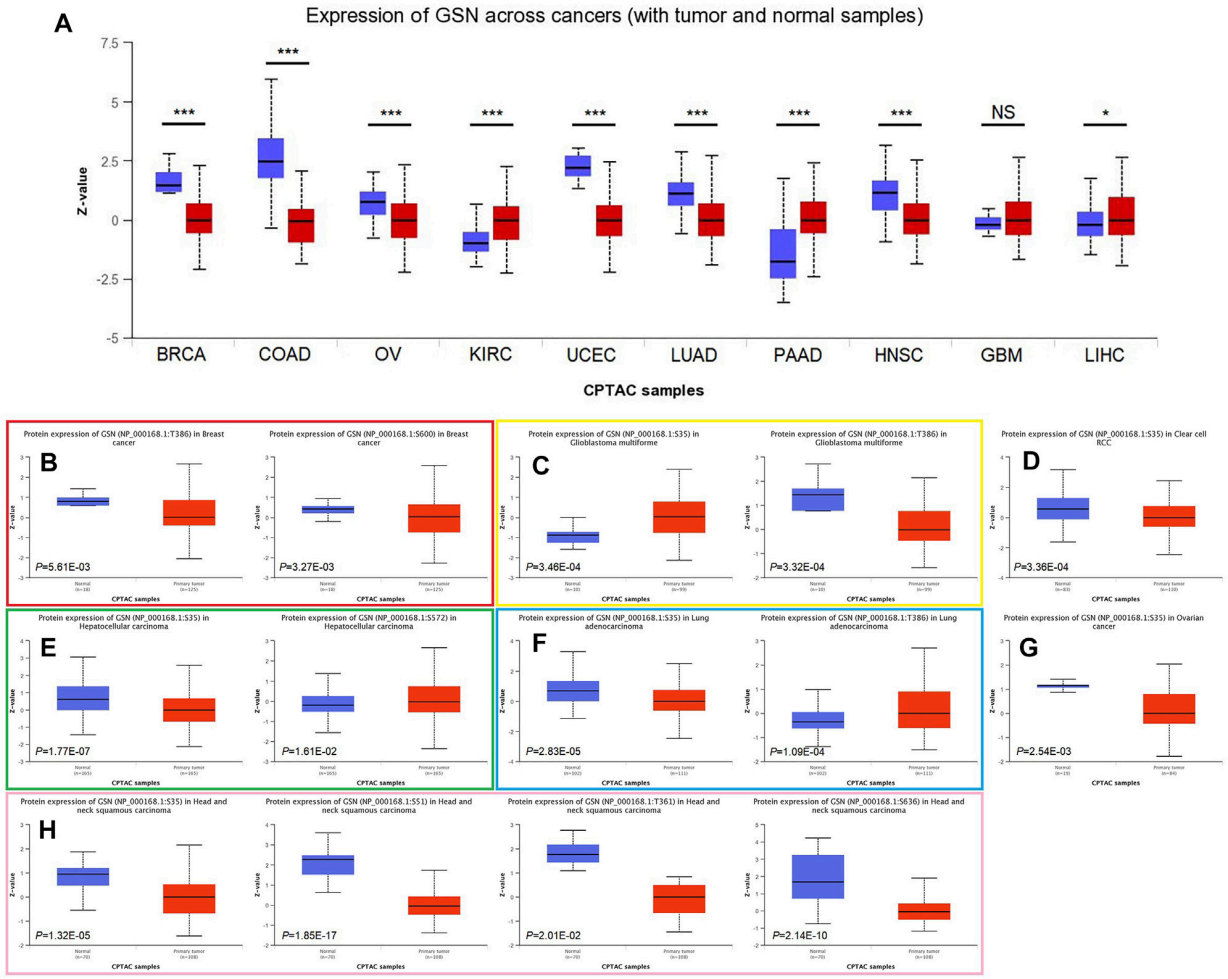
Differences in GSN protein content and phosphorylation levels in pan-carcinoma. **(A)** Differences in GSN protein content in pan-carcinoma, including BRCA, COAD, OV, KIRC, UCEC, LUAD, PAAD, HNSC, GBM, and LIHC. Phosphorylation levels of GSN proteins varied in BRCA **(B)**, GBM **(C)**, KIRC **(D)**, LIHC **(E)**, LUAD **(F)**, OV **(G)**, and HNSC **(H)**. (*p < 0.05, ***p < 0.001, NS: *p* > 0.05).

We explored protein expression and phosphorylation levels of GSN using the UALCAN database. We found lower GSN protein expression in BRCA, COAD, OV, UCEC, LUAD and HNSC compared to healthy tissues, as well as higher GSN protein expression in KIRC, PAAD and LIHC, but no difference in GSN protein expression in GBM. Further, we utilized the HPA database to observe immunohistochemical photos to measure protein expression levels of GSN (Figure 7A). We observed that the protein expression of GSN in BLCA, BRCA, CESC, COAD, OV and UCEC was significantly reduced contrasted with that of the matching healthy tissue (Supplementary Figure S2). The protein expression of GSN in LGG, LIHC and PAAD was significantly higher compared to the matching healthy tissue. Next, we explored the phosphorylation levels of GSN protein. We observed variations in GSN protein phosphorylation levels in seven malignancies: BRCA, GBM, KIRC, LIHC, LUAD, OV, and HNSC (Figures 7B–H). Among them, S35 was the most crucial phosphorylation modification site, and except for GBM, the phosphorylation level of S35 in other cancers was decreased compared to that of healthy tissues (Figures 7C–H). In HNSC, we found that the GSN protein had the most phosphorylation modification sites, and the phosphorylation was reduced compared to that of healthy tissue (Figure 7H).

**FIGURE 8:**
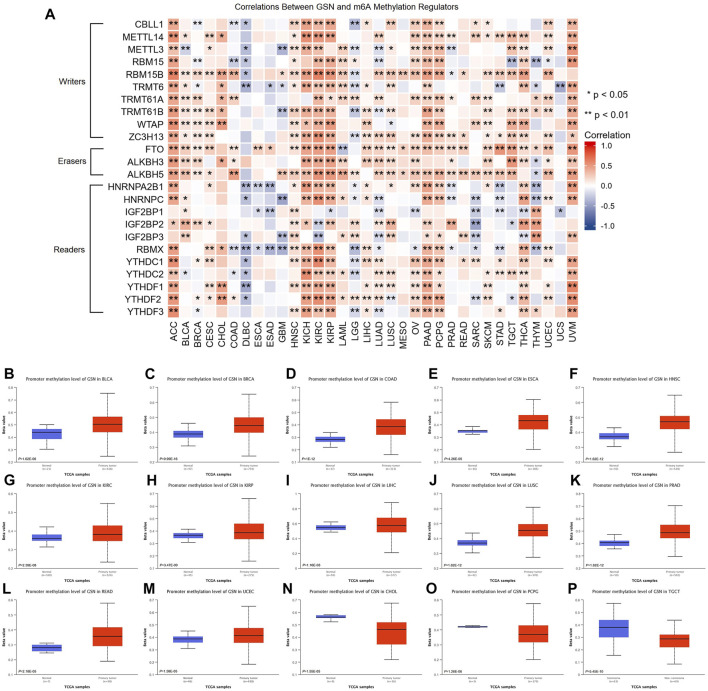
Epigenetic methylation analysis of GSN. **(A)** The correlation between the expression of GSN mRNA and m6A methylation regulatory factors in multiple cancers. Differential promoter methylation level (beta values) of GSN in normal tissues and tumors based on UALCAN, including BLCA **(B)**, BRCA **(C)**, COAD **(D)**, ESCA **(E)**, HNSC **(F)**, KIRC **(G)**, KIRP **(H)**, LIHC **(I)**, LUSC **(J)**, PRAD **(K)**, READ **(L)**, UCEC **(M)**, CHOL **(N)**, PCPG **(O)**, and TGCT **(P)**.

We examined the link between GSN mRNA expression and m6A methylation controllers in several tumors because m6A methylation plays a significant part in carcinogenesis and development. In total, 24 essential m6A methylation controllers were chosen: 10 writers (CBLL1, METTL14, METTL3, RBM15, RBM15B, TRMT6, TRMT61A, TRMT61B, WTAP, ZC3H13), 3 erasers (FTO, ALKBH3, ALKBH5), and 11 readers (HNRNPA2B1, HNRNPC, IGF2BP1, IGF2BP2, IGF2BP3, RBMX, YTHDC1, YTHDC2, YTHDF1, YTHDF2, YTHDF3). The heatmap showed that in ACC, KICH, KIRC, KIRP, PAAD, PCPG, THCA, as well as UVM, GSN expression was positively linked to the expression of many m6A methylation regulators (Figure 8A). Moreover, we contrasted promoter methylation contents of GSN in healthy and tumor tissues. The findings declared that the GSN promoter was hypermethylated in several malignancies: BLCA, BRCA, COAD, ESCA, HNSC, KIRC, KIRP, LIHC, LUSC, PRAD, READ and UCEC (Figures 8B–M). In contrast, the GSN promoter was hypomethylated in CHOL, PCPG, and TGCT contrasted with healthy tissues (Figures 8N–P).

Certain DNA methylations play a massive role in tumor immunogenicity (Hogg et al., 2020). Subsequently, we employed the TISDIB database to explore the connection between GSN methylation patterns and immune cell infiltration. Moreover, the heat map exhibited that GSN methylation levels were adversely linked to the infiltration degree of most immunity cells in ACC, BLCA, BRCA, COAD, GBM, KIRP, LGG, LIHC, PCPG, and PRAD, while were positively linked to the infiltration degree of most immune cells in CESC, ESCA, HNSC, LUAD, LUSC, and SARC (Supplementary Figure S3A). We utilized the GSCA database to examine the association between GSN methylation patterns and GSN mRNA expression and their influence on the outcome of cancer patients. Accordingly, GSN methylation levels were adversely related to GSN mRNA expression in most cancers except for CHOL and DLBC (Supplementary Figure S3B). Moreover, the hypomethylation level of GSN was an adverse variable influencing the outcome of LGG and BLCA patients (Supplementary Figure S3C).

In conclusion, the GSN protein exhibited low phosphorylation levels, and the GSN promoter exhibited hypermethylation and affected immune cell invasion and patient outcomes in most cancers.

### 3.5 Genetic changes characteristics of GSN

**FIGURE 9:**
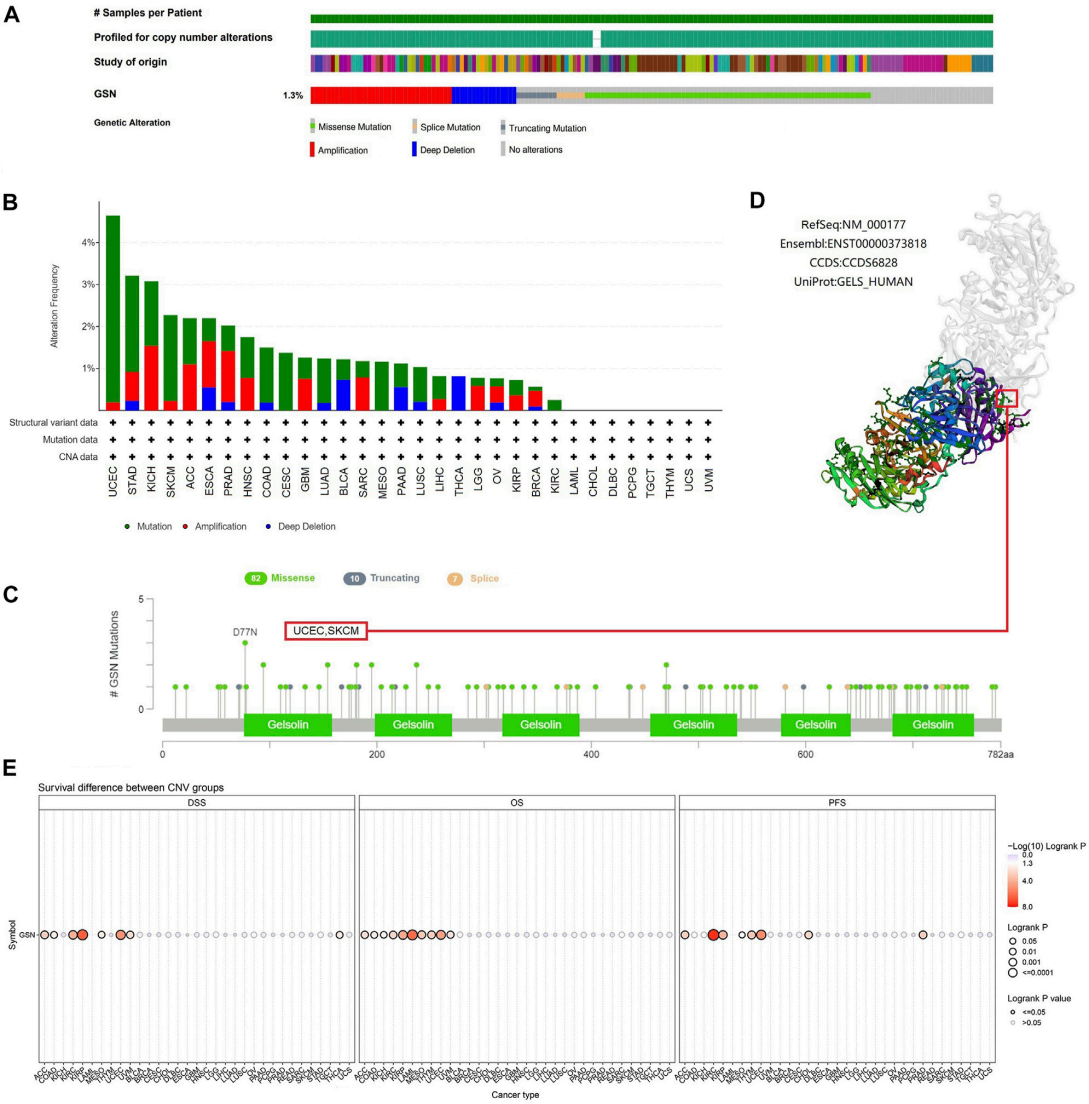
Mutated features of GSN in different tumors. **(A)** Summary of changes in GSN expression in different tumors. **(B)** Bar plot of GSN alteration frequency and types across different cancer types. **(C)** The landscape of GSN mutation with the location, types, and number and their relationship with protein domains. **(D)** Some GSN mutations are shown on the 3D structure of the protein. **(E)** Correlation between CNV in GSN and prognosis in cancer patients.

Cancer is driven by many genetic changes, some of which are potential molecular therapeutic targets (Ben-David and Amon, 2020). Novel therapeutics targeting highly mutated transcription factor TP53 gene products have performed well in pan-carcinoma (Stephenson Clarke et al., 2022). Therefore, we explored its genetic alterations to investigate whether GSN can be used as a target for molecular therapy. We found that 139 of the 10,443 samples (1.3%) developed GSN mutations, and missense mutations were the most common in GSN (Figure 9A). Among all mutations, 45.36% of mutations belonged to missense substitution, and 19.20% of mutations belonged to synonymous substitution (Supplementary Figure S4B). Additionally, the most dominant SNV categories were G > A (35.53%), followed by C > T (30.92%) (Supplementary Figure S4C). The five cancer types that had the greatest mutation frequency were: UCEC (4.64%), STAD (3.21%), KICH (3.08%), SKCM (2.27%), and ACC (2.20%, Figure 9B). D77N in the gelsolin-like 1 domain was the greatest site with mutations, which occurred in two patients with UCEC and one with SKCM (Figure 9C). We exhibited it in the 3D structure of GSN protein (Figure 9D). GSN changes were significantly linked to higher OS (*p* = 0.0340) and PFS (*p* = 0.0107) in UCEC patients (Supplementary Figures S4E–F).

Subsequently, we utilized the GSCA database to study the association between GSN mutation and GSN mRNA expression and GSN mutation and outcome of cancer patients. CNV mutations in GSN were adverse factors affecting the outcome of ACC, KIRC, KIRP, MESO and UCEC patients (Figure 9E). CNV pie chart results showed that heterozygous amplification and heterozygous deletion occurred in most cancers. In contrast, rare homozygous amplification occurred mainly in ACC, PRAD and SARC, and rare homozygous deletion occurred mainly in BLCA, READ and THCA (Supplementary Figure S4A). We identified a positive association between GSN mutations and GSN mRNA expression in LUSC, BLCA, HNSC, OV, ESCA, UCEC, KICH, SKCM, BRCA, LUAD, SARC and PAAD (Supplementary Figure S4D). Genetic alterations in GSN occurred in most cancers and were linked to the outcome of cancer patients.

### 3.6 GSN is related to immune invasion and immune response in pan-cancer

**FIGURE 10:**
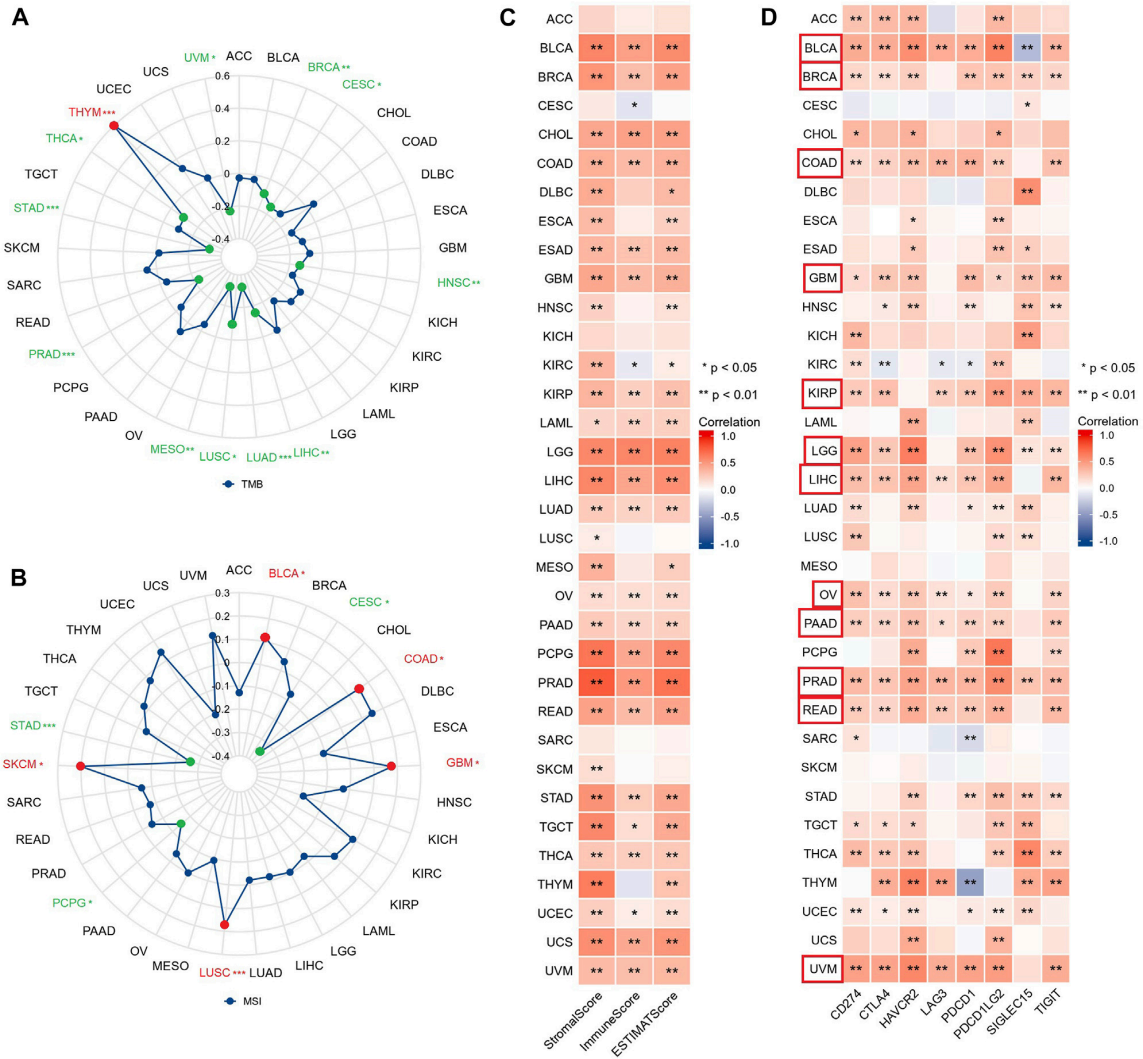
GSN expression is associated with TMB, MSI, TME, and immune checkpoints in 33 cancer types. Relationship between GSN expression and TMB **(A)**, MSI **(B)** in 33 cancers. **(C)** Relationship between GSN expression and StromalScore, ImmuneScore, and ESTIMATEScore in 33 cancers. **(D)** Relationship between GSN expression and immune checkpoint expression in 33 cancers.

TMB and MSI can respond to the state of immunotherapy as predictive biomarkers of tumor treatment (Filipovic et al., 2020). The radar chart revealed that GSN expression was adversely linked to TMB in 11 cancer types: BRCA, CESC, HNSC, LIHC, LUAD, LUSC, MESO, PRAD, STAD, THCA, and UVM, but only positively associated with TMB of THYM (Figure 10A). Moreover, GSN expression was positively linked to MSI in BLCA, COAD, GBM, LUSC, and SKCM. In contrast, GSN expression was adversely connected to MSI in CESC, PCPG, and STAD (Figure 10B). We measured the connection between stromal and immunological scores and GSN expression in pan-carcinoma. We found that GSN expression was positively linked to StromalScore, ImmuneScore, and ESTIMATEScore in BLCA, BRCA, CHOL, COAD, ESAD, GBM, KIRP, LAML, LGG, LIHC, LUAD, OV, PAAD, PCPG, PRAD, READ, STAD, TGCT, THCA, UCEC, UCS and UVM. In contrast, GSN expression was positively related to StromalScore and ESTIMATEScore in DLBC, ESCA, HNSC, KIRC, MESO, and THYM (Figure 10C).

We studied the connection between GSN and immune checkpoints. GSN expression was positively linked to the expression of many immune checkpoints in BLCA, BRCA, COAD, GBM, KIRP, LGG, LIHC, OV, PAAD, READ, and UVM (Figure 10D). Subsequently, we evaluated the connection between GSN expression and immune-related gene expression, including 43 immune activation-related genes, 22 immunosuppression-related genes, 21 MHC-related genes, 41 chemokines, and 18 chemokine receptors. GSN expression was positively associated with many immune-related genes in BLCA, BRCA, COAD, KIRP, LGG, LIHC, OV, PAAD, PCPG, PRAD, READ, TGCT, THCA, THYM, and UCM (Supplementary Figures S5A–E). In conclusion, in most cancers, GSN expression was significantly associated with the immune score, immune checkpoints, and immune-related genes.

**FIGURE 11:**
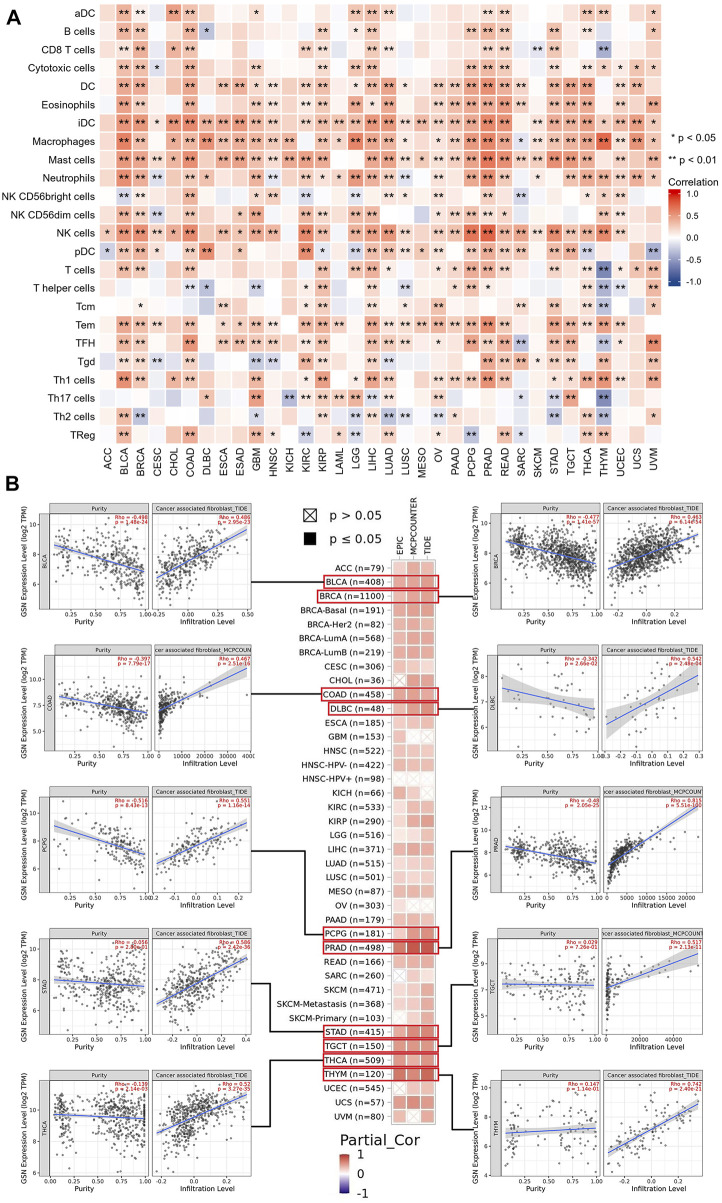
Associations between immune cell infiltration levels and GSN expression in pan-cancer. **(A)** The correlation of GSN expression and immune infiltration using the ssGSEA algorithm. **(B)** GSN expression correlation analysis with immune infiltration of CAF cells based on Timer2.0 database, scatter plots including BLCA, BRCA, COAD, DLBC, PCPG, PRAD, STAD, TGCT, THCA, and THYM.

TIICs were a crucial component of the tumor microenvironment (TME) and were closely related to the aggressiveness of cancer. We employed the ssGSEA method to evaluate the association between GSN expression and the 24 immune cells infiltration level. The heat map findings indicated that GSN expression was positively linked to the invasion degree of most immune cells in most cancers like BLCA, BRCA, COAD, KIRP, LIHC, LUAD, PRAD, READ, THCA, and the degree of invasion of some of these immune cells was significantly linked to GSN expression, such as DC cells, immature DC cells (iDC), macrophages, mast cells, neutrophils, eosinophils, NK cells, T effector memory cells (Tem), T follicular helper cells (TFH, Figure 11A). Furthermore, we examined the link between GSN expression levels and various tumor immunological cell infiltration utilizing EPIC, MCP-COUNTER, CIBERSORT, and TIDE algorithms through the Timer2.0 database. In many cancers, GSN expression was positively linked to the CAFs infiltration level, with GSN expression significantly linked to the degree of CAFs infiltration in BLCA (r = 0.486), BRCA (r = 0.463), COAD (r = 0.467), DLBC (r = 0.542), PCPG (r = 0.551), PRAD (r = 0.815), STAD (r = 0.586), TGCT (r = 0.517), THCA (r = 0.520), and THYM (r = 0.742) (Figure 11B). In many cancers, GSN expression was significantly linked to mDCs invasion (Supplementary Figure S6A). We also found that in THYM, GSN expression was adversely linked to the infiltration level of CD8+ T cells, CD4+ T cells, Tregs, and mDCs, and positively related to the infiltration level of neutrophils, monocytes, macrophages, and NK cells (Supplementary Figure S6A). We studied the association between GSN expression and biomarkers of CAFs and mDCs. The findings declared that GSN expression was positively linked to the biomarker expression of CAFs in nearly all malignancy forms (Supplementary Figure S6B). GSN expression was positively linked to the expression of all biomarkers of mDCs in BLCA, BRCA-LumA, LIHC, PRAD, STAD, and TGCT (Supplementary Figure S6C). In most cancers, GSN expression was positively connected to the invasion degree of multiple immunological cells, particularly CAFs and mDCs.

### 3.7 Functional enrichment analysis of GSN

**FIGURE 12:**
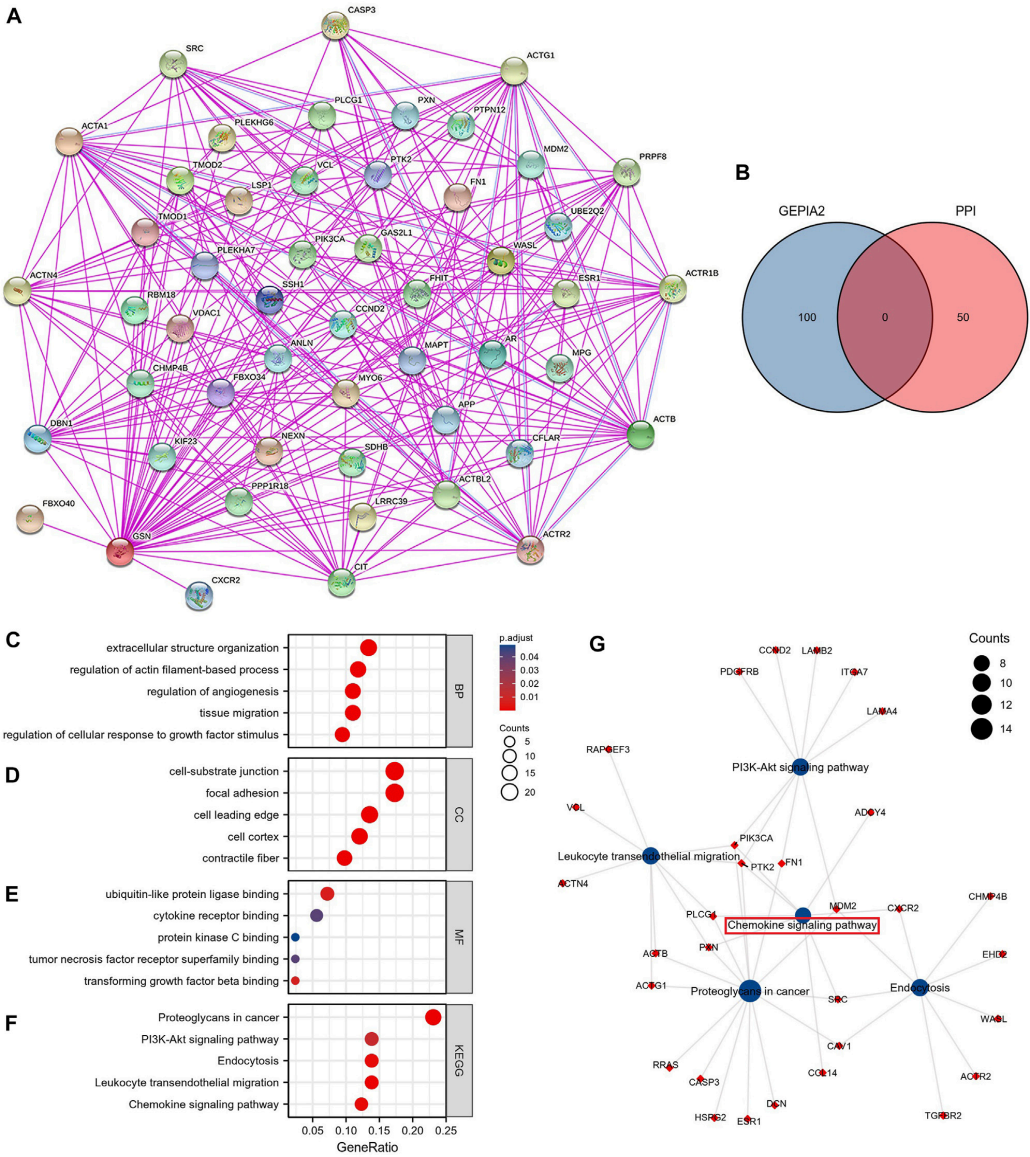
GSN-related genes, interacting proteins and functional enrichment analysis. **(A)** PPI Network for GSN. **(B)** The intersection of GSN-binding and interacting genes after selection by Venn diagram analysis. GO analyses, including biological process **(C)**, cellular component **(D)**, molecular function **(E)**, and KEGG pathway **(F)**. **(G)** Visual network of KEGG analyses.

To fully comprehend the possible molecular pathways of GSN in tumor initiation and establishment, we explored the enrichment analysis of GSN co-expressed genes. We used the GEPIA2 database to obtain the first 100 GSN co-expressed genes (Supplementary Table S2). The 50 GSN-binding proteins acquired from the STRING database were utilized to build a PPI network (Figure 12A). The venn plot shows no genes in common (Figure 12B). Subsequently, the first 100 GSN co-expressed genes and 50 GSN-binding proteins were involved in the functional enrichment analysis. Eventually, 357 GO categories were noticed, including 337 biological processes (BP), 55 cellular components (CC), and 44 molecular functions (MF), aside from 49 KEGG pathways (Supplementary Table S3). We presented the top five cancer-related items in each GO entry. The results showed that BP was mainly involved in the extracellular structure organization, actin filament-based process regulation, angiogenesis, tissue migration, and cellular response to growth factor stimulus (Figure 12C). The CC was mainly enriched in the focal adhesion, cell-substrate junction, cell leading edge, cell cortex, and contractile fiber (Figure 12D). The MF contained ubiquitin-like protein ligase binding, cytokine receptor binding, protein kinase C binding, tumor necrosis factor receptor superfamily binding, and transforming growth factor beta binding (Figure 12E). KEGG pathway analysis found that proteoglycans may mediate GSN in cancer, PI3K-Akt signaling pathway, endocytosis, leukocyte transendothelial migration, and chemokine signaling pathway (Figure 12F). We visualized the KEGG pathway and found that the Chemokine signaling pathway had the most overlapping genes, suggesting that the Chemokine signaling pathway may be a critical GSN-mediated pathway (Figure 12G).

**FIGURE 13:**
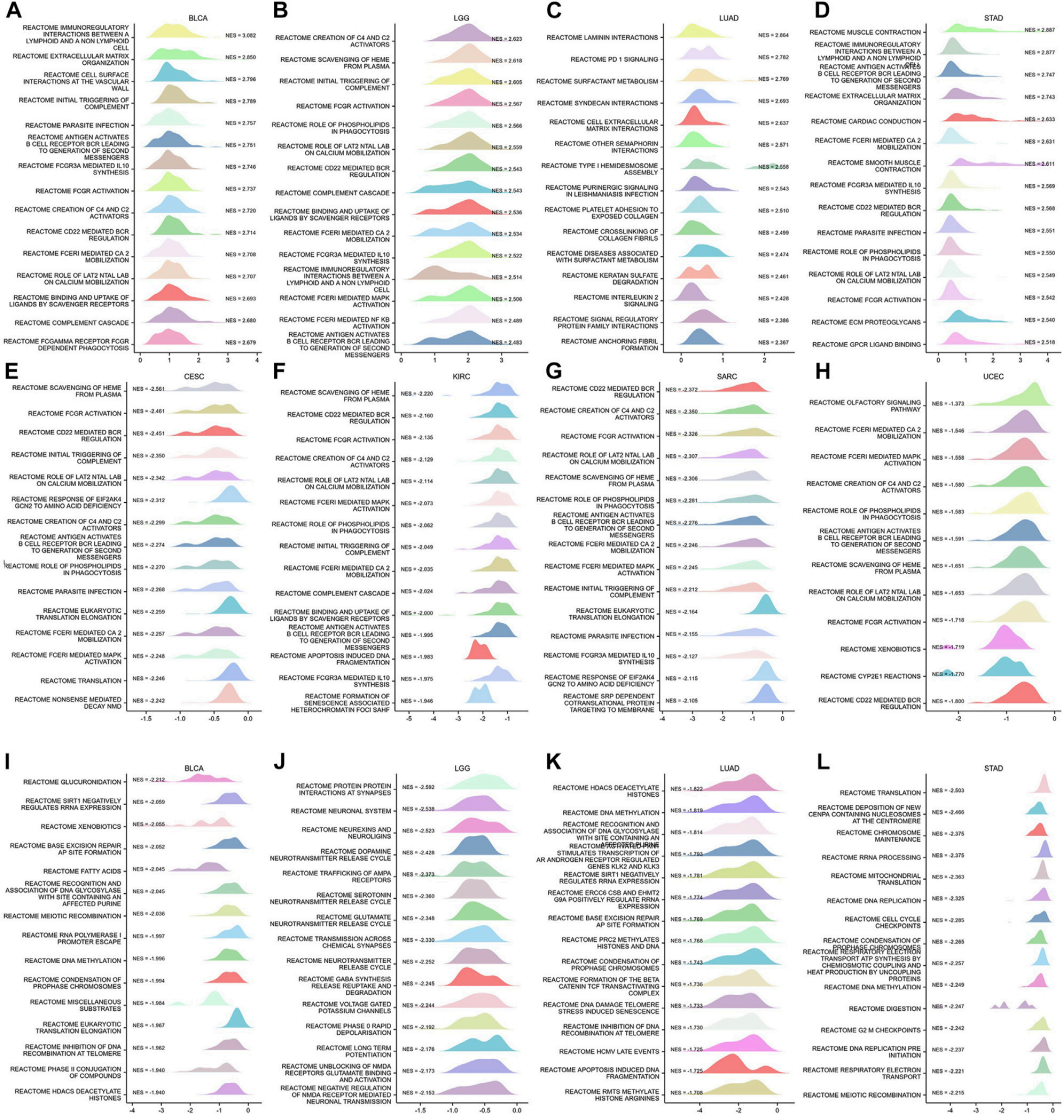
GSEA functional enrichment analysis of GSN in 8 cancers. In BLCA **(A)**, LGG **(B)**, LUAD **(C)**, and STAD **(D)**, the first 15 reaction pathways were positively correlated with GSN expression. In CESC **(E)**, KIRC **(F)**, SARC **(G)**, UCEC **(H)**, BLCA **(I)**, LGG **(J)**, LUAD **(K)**, and STAD **(L)**, the first 15 reaction pathways negatively correlated with GSN expression.

To identify the possible mechanisms for GSN involvement in pan-cancer, we subsequently performed GSEA analysis according to the reactome pathway database. Our analysis included eight cancer types: BLCA, LGG, LUAD, and STAD, whose outcome was positively linked to GSN expression, and CESC, KIRC, SARC, and UCEC, prognosis was negatively correlated with GSN expression. Our GSEA results showed that among cancer types with a favorable outcome associated with GSN expression, genes positively related to GSN expression were mainly enriched in the immune-associated reactome pathway (Figures 13A–H). Similarly, in cancer types with a prognosis negatively associated with GSN expression, genes adversely linked to GSN expression were also mainly enriched in the immune-related reactome pathway. The enriched pathways mainly included immunomodulatory interactions between lymphocytes and non-lymphocytes, activation of complement signaling pathways, antigen activation of B cell receptors BCR resulting in the production of second messengers, and Cd22-mediated BCR regulation. Furthermore, the enriched pathways suggested that GSN mediated the activation of FCGR pathway and FCERI pathway, mediated downstream IL-10 synthesis, Ca2 mobilization, MAPK activation and other biological functions (Figures 13A–H). Moreover, we found in BLCA, LGG, LUAD and STAD that genes negatively associated with GSN expression are mainly enriched in DNA methylation, protein post-translational modification, mediating cell cycle and other functions, including histone deacetylation and methylation, cell cycle checkpoints, G2M checkpoints and other reactome pathways (Figures 13I–L).

In conclusion, we inferred that GSN played an essential role in cancer primarily by influencing immune-related pathways and regulating biological functions such as DNA methylation.

## 4 Discussion

This research mainly investigated the influence of GSN on carcinogenesis and progression and its molecular mechanism, including cancer cell proliferation, metastasis, and the means of GSN-mediated EMT. We are the first to examine the involvement of GSN in pan-cancer. The findings revealed that GSN expression varied across 33 malignancies, and GSN expression was significantly elevated in 11 cancer forms and reduced significantly in 17 cancer forms. Notably, we found more pronounced differences in GSN in the early stages of BLCA and KIRC, suggesting that GSN may have the potential to be an early diagnostic marker in both tumor types. In most cancers, we found differences in GSN expression in various subtypes. It should be noted that GSN tends to be highest expressed in the C3 immune subtype and lowest expressed in the C4 immune subtype. Accordingly, the C3 immune subtype has the best survival outcome, whereas the C4 immune subtype has the worst survival outcome, which is consistent with previous studies (Thorsson et al., 2018).

Next, our study determined the predictive value of GSN in pan-cancer and discovered that GSN also possessed various predictive significances in various cancer forms. Notably, GSN was highly expressed in LAML and LGG, while patients who showed elevated GSN expression showed worse outcomes. GSN was low in BRCA, LUAD, CESC, and UCEC, while patients with low GSN expression had a poor prognosis. Upregulated UHRF1 silences GSN to suppress the death of early cervical cancer cells in CESC (Lee et al., 2020). Breast cancer is the first cancer afflicting women worldwide and has replaced lung cancer as the most common cancer worldwide (Sung et al., 2021). TGF-β1 upregulation can increase GSN expression, inhibit cancer cell growth and progression, as well as promote cancer cell migration (Chen et al., 2015). In OV, it has been reported that the OS and PFS of GSN-positive patients were significantly lower than GSN-negative patients, which may be because high GSN expression conferred chemical resistance to cancer cells by altering GSN-FLICE-like inhibitory protein (FLIP)-Itch interaction. Pgsn can be released and transmitted through exosomes (Ex-pGSN), Autocrine upregulation of HIF1α-mediated chemical resistance (Abedini et al., 2014; Asare-Werehene et al., 2020). However, we unobserved such results in OV, and we speculated that this was due to the large number of advanced cancer samples included in the above studies and the insufficient sample size. Wu et al. (2022) declared that GSN expression was dropped in STAD, reduced GSN expression was linked to reduced survival in patients with STAD, and GSN expression was significantly related to STAD tumor purity and degree of DC cell invasion. However, we discovered that GSN high expression was a negative variable affecting the outcome of STAD patients. Therefore, the difference in selected data and the threshold difference in split patients might cause a difference in results. So far, the researchers have unpassed the experimental report, and more data and functional trials are still needed to verify in the future. We observed GSN expression to be an independent predictive variable for BLCA, LAML, LGG, STAD, and SARC, which greatly enriched traditional predictive models, but no related studies have been reported. In CESC, GSN was used as a characteristic gene to construct an effective tool for predicting OS (Li et al., 2022). Finally, GSN is a promising marker for future cancer management.

Next, we examined the diagnostic significance of GSN in pan-cancer. We found that GSN had the best diagnostic efficacy in BRCA, which was validated using serum from breast cancer patients (AUC = 0.947). Serum GSN (AUC = 0.932) levels are superior to common tumor biomarkers, carcinoembryonic antigen (CEA), or carbohydrate antigen 19–9 (CA199) for colon cancer (Chen et al., 2019b). Randall et al. (Brock et al., 2012) reported that for the training set containing 321 COAD samples, 6 serum proteins containing GSN achieved a diagnostic value of AUC = 0.9003 for COAD and AUC = 0.8989 in the validation set containing 110 samples. Further, in esophageal adenocarcinoma, the diagnostic efficacy AUC of serum GSN alone was stabilized at around 0.7 (Shah et al., 2018). The diagnostic value of serum GSN protein alone for pancreatic cancer in diabetic patients was also good (AUC = 0.75) (Peng et al., 2020). In conclusion, GSN may have good predictive potential in many cancer types. However, few relevant studies exist, and more extensive investigations are further required in the future to explore the feasibility of GSN as a diagnostic marker.

Epigenetic modifications play a vital role in tumors through various mechanisms (Sun et al., 2022). VEERLE et al. (De Corte et al., 1999) identified Tyr438 as the most prominent site of GSN phosphorylation by mass spectrometry. We found that S35 was the most common phosphorylation modification site of GSN in most cancers, but whether it is a functional site needs further research. m6A methylation was strongly linked to cancer cell growth, metastasis, immune response and other processes and affected the sensitivity and resistance of anti-cancer treatment drugs (Lan et al., 2021). Therefore, we indirectly explored the level of GSN methylation modification in pan-carcinoma and its role. In most malignancies, GSN expression is positively correlated with m6A methylation-related gene expression, therefore, we hypothesize that GSN has lower levels of m6A methylation, and the GSN promoter was hypermethylated. In addition, in most cancers, the level of DNA methylation of GSN is negatively correlated with the invasion of immune cells in TME. There are literature reports on raised GSN expression patterns in CESC cells subjected to DNA-hypomethylating agent 5-aza-2′-deoxycytidine (Lee et al., 2020). Tumor-associated macrophages (TAMs) were co-cultured with gastric cancer cells, and DNA methyltransferase 1 (DNMT1) expression increased; however, GSN expression decreased in gastric cancer cells (Wang et al., 2017). In breast cancer, GSN downregulation was triggered *via* hypermethylation of essential DNA methylation sites. A risk score model with excellent prognosis reliability was developed using three methylation probes based on CAV2 and GSN genes (Cao et al., 2022). In conclusion, epigenetic modifications of GSN played an essential role in pan-cancer, but more functional experimental verification mechanisms are needed in the future.

Missense mutations are the most frequent among GSN mutations, the most occurring GSN mutations in UCEC, and D77N in the gelsolin-like 1 domain is the site with the most frequent mutations. In some cancers, the CNV status of GSN was positively correlated with GSN expression and was an adverse factor affecting patient outcomes. We are the first to reveal the significance of GSN mutations in pan-cancer, but more research is required to identify the pathway.

With the advent of immunotherapy, cytokine and immune checkpoint inhibitor (ICI) therapies have gradually proven as medications for several malignancies (Havel et al., 2019). TMB and MSI are predictors of the anti-tumor efficacy of ICIs (Xu et al., 2020). Higher TMB and MSI mean a better response to ICI and a better prognosis for cancer patients (Chen et al., 2019a; Samstein et al., 2019). Our study observed that GSN expression was adversely linked to TMB in LIHC, while GSN was highly expressed in LIHC. GSN expression was positively related to MSI in BLCA, COAD, LUSC, as well as SKCM, whereas GSN was low expressed in these cancers. Therefore, we speculated that GSN is responsible for the low TMB and MSI in the above cancers, predicting that GSN may play a role in immunotherapy. Moreover, in BLCA, BRCA, COAD, GBM, KIRP, LGG, LIHC, OV, PAAD, READ, and UVM, GSN expression was positively linked to immunological scores, most immune checkpoints, and expression of immune-related genes. According to our results, GSN could regulate cancer immunity, and targeting GSN might become a new strategy for tumor immunotherapy.

No correlation analysis has involved GSN and the TME. We observed that GSN expression was positively correlated with the infiltration level of most immunity cells, like DC cells, macrophages, NK cells, Tem, TFH and other immune cells involved in anti-tumor immune effects, and CAF involved in tumor immune evasion or suppression. GSN was low expressed in most cancers, so we speculated that GSN is mainly involved in immune effects in the TME through anti-tumor immune invasion rather than immune escape or immunosuppression. These immune cells can participate in tumor immunity through various mechanisms, including secreting multiple cytokines and chemokines and antigen presentation, mediating the enrollment and functional development of innate and adaptive immunity cells (Ghesquiere et al., 2014; Monteran and Erez, 2019; Shimasaki et al., 2020). Our KEGG analysis showed that GSN might mediate proteoglycans in cancer, PI3K-Akt signaling pathway, endocytosis, leukocyte transendothelial migration, and chemokine signaling pathway. The chemokine signaling pathway was identified as the most critical pathway mediated by GSN. In addition to regulating inflammatory responses, promoting cancer cell metastasis, and regulating apoptosis, glycosylation changes may also regulate inflammatory reactions (Reily et al., 2019).

Similarly, our GSEA analysis showed that in cancer types where GSN expression was negatively associated with prognosis, GSN was positively correlated with immune function. Among the cancer types whose expression was positively associated with prognosis, GSN was negatively correlated with immune function. Although GSN had different effects on the outcome of patients with several malignancies, they all showed the same immune trend; GSN negatively regulated the prognosis of cancer patients by mediating the immune effect in pan-cancer. In cancer forms in which GSN expression was adversely associated with patient outcomes, GSN was negatively correlated with processes such as DNA methylation and cell cycle, reconfirming our previous discussion about the effects of GSN methylation and GSN expression on cell proliferation, invasion, and migration.

## 5 Conclusion

We conducted the first pan-cancer study of GSN, including expression, prognostic and diagnostic, epigenetics, methylation, immunoassay, and enrichment analyses, indicating that GSN was a potential therapeutic biomarker for malignancy. However, this research has certain restrictions, like a small sample size and lack of experimental validation. In the future, the research sample should be expanded to study the detailed carcinogenic mechanism of GSN in pan-carcinoma through *in vitro* and *in vivo* experiments.

## Data Availability

The original contributions presented in the study are included in the article/Supplementary Material, further inquiries can be directed to the corresponding authors.
