# Supplementary material for: Gelsolin: A comprehensive pan-cancer analysis of potential prognosis, diagnostic, and immune biomarkers
Source: Front Genet. 2023 Mar 23;14:1093163. doi: 10.3389/fgene.2023.1093163 (PMC10076574; doi:10.3389/fgene.2023.1093163)
Supplement: Supplementary file 1 [file Table1.DOCX]

**Supplementary materials**

**Supplementary materials**

Additional material for this article can be found in the Supplementary Graphics and Table Legend module.


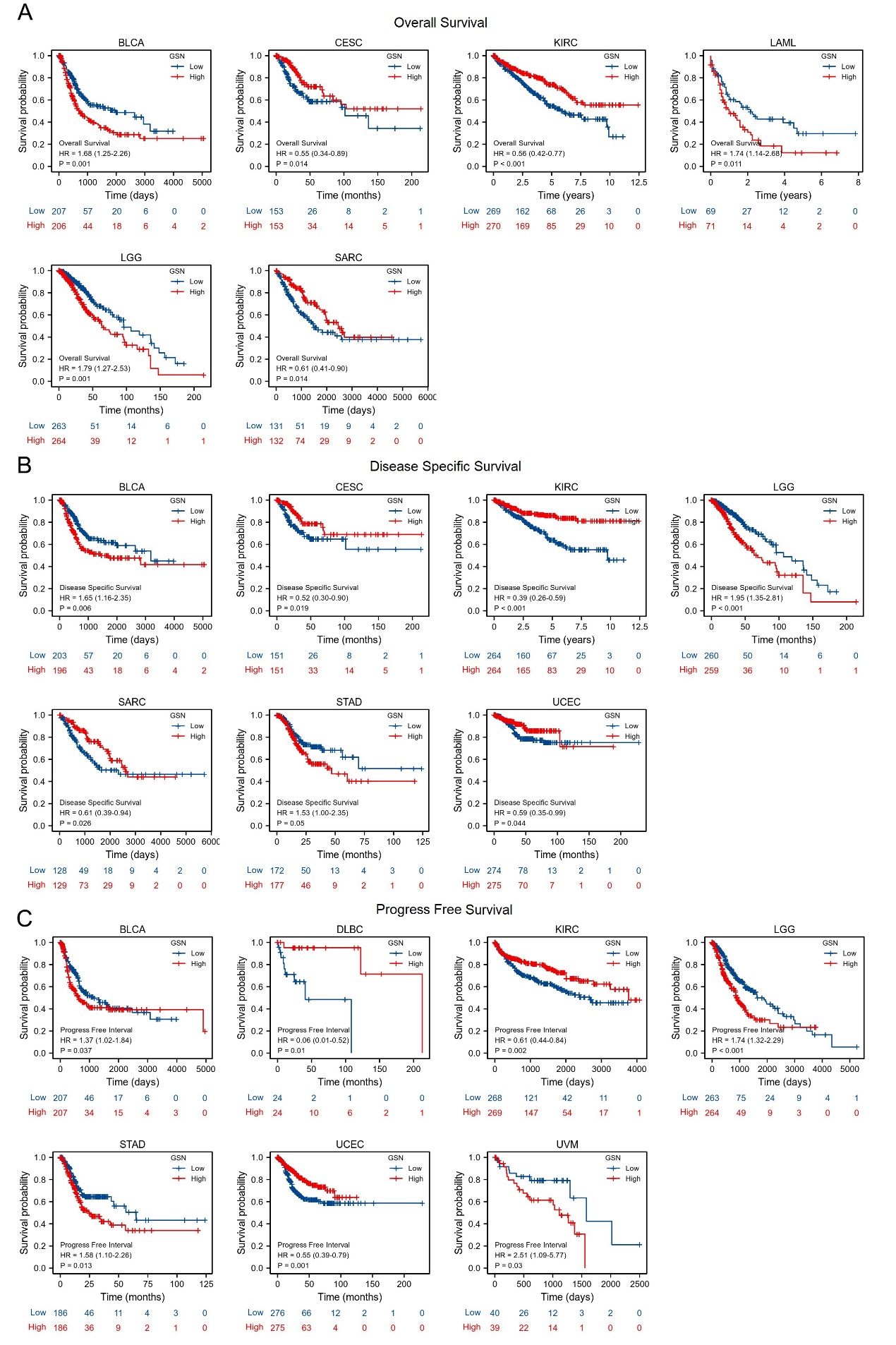


**Supplementary Figure S1.** The KM curve shows in detail the association between GSN expression and prognosis in cancer patients. **(A)** OS in BLCA, CESC, KIRC, LAML, LGG, and SARC. **(B)** DSS in BLCA, CESC, KIRC, LGG, SARC, STAD, and UCEC. **(C)** PFS in BLCA, DLBC, KIRC, LGG, STAD, UCEC, and UCM.


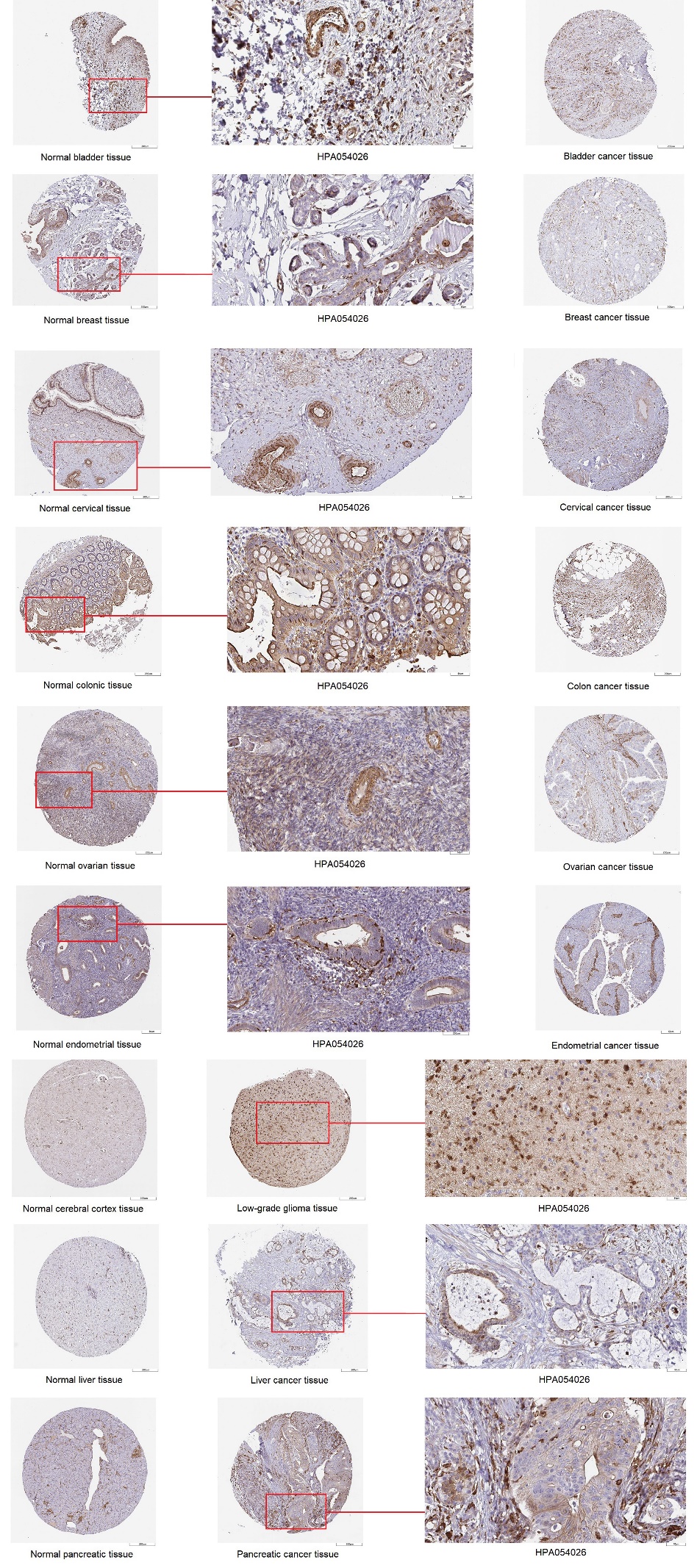


**Supplementary Figure S2.** GSN protein expression in immunohistochemical images of normal and tumor groups, including BLCA, BRCA, CESC, COAD, OV, UCEC, LGG, LIHC, and PAAD.


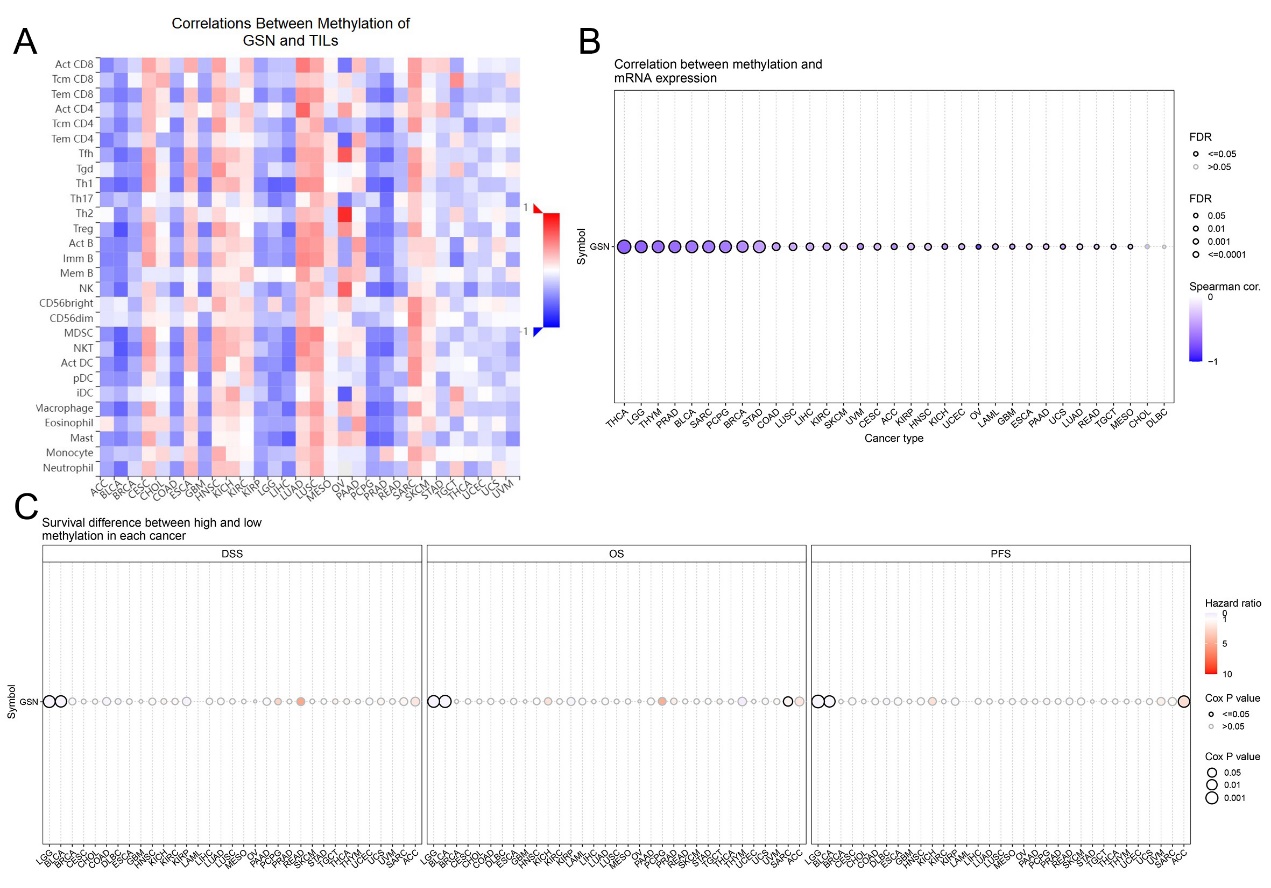


**Supplementary Figure S3.** Epigenetic methylation analysis of GSN. **(A)** Association between GSN methylation levels and immune cell invasion in pan-carcinoma based on TISIDB database. **(B)** Relationship between GSN methylation level and GSN mRNA expression. **(C)** Effect of GSN methylation levels on prognosis in cancer patients.


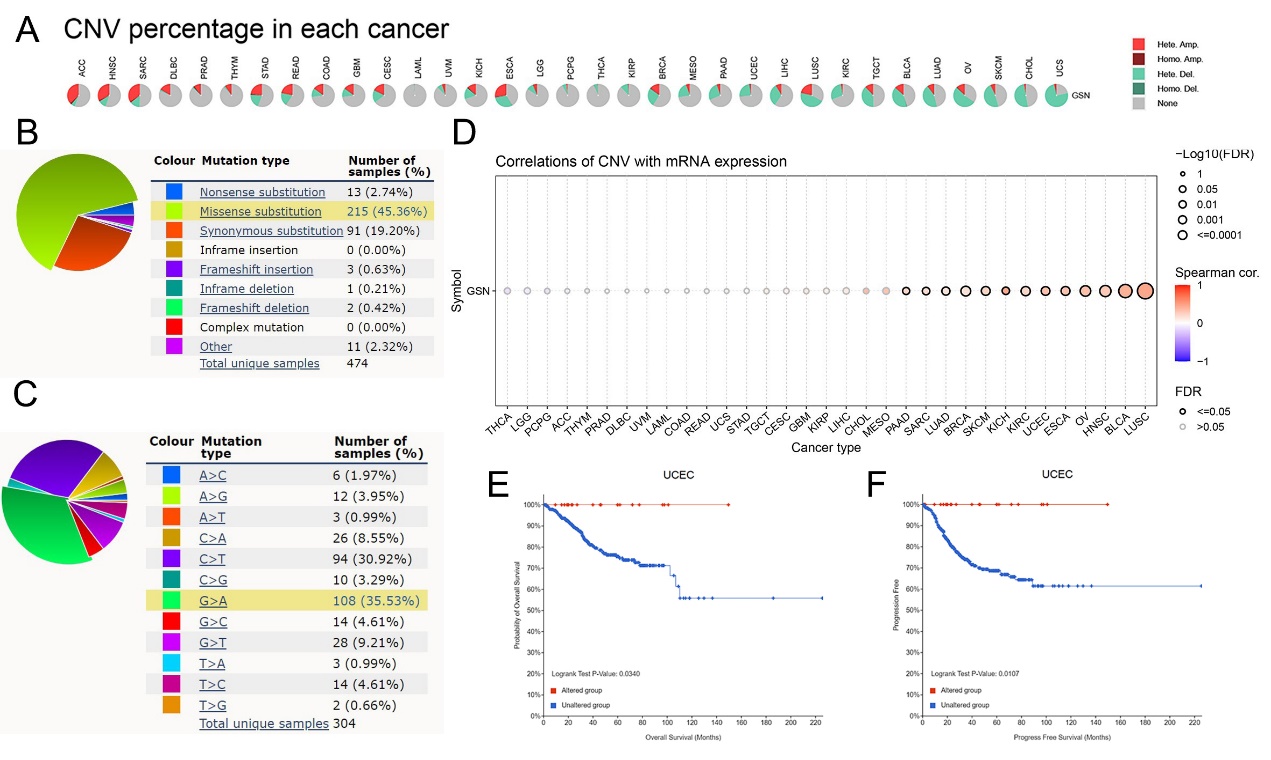


**Supplementary Figure S4.** Mutation analysis of GSN. **(A)** GSN copy number variant (CNV) percentage in each cancer. **(B)** The main mutation type of TUBA1B. **(C)** The main type of single nucleotide variant (SNV) of GSN. **(D)** Correlation between GSN expression and CNV. Correlation between CNV in GSN and prognosis in UCEC patients, including OS **(E)** and PFS **(F)**.


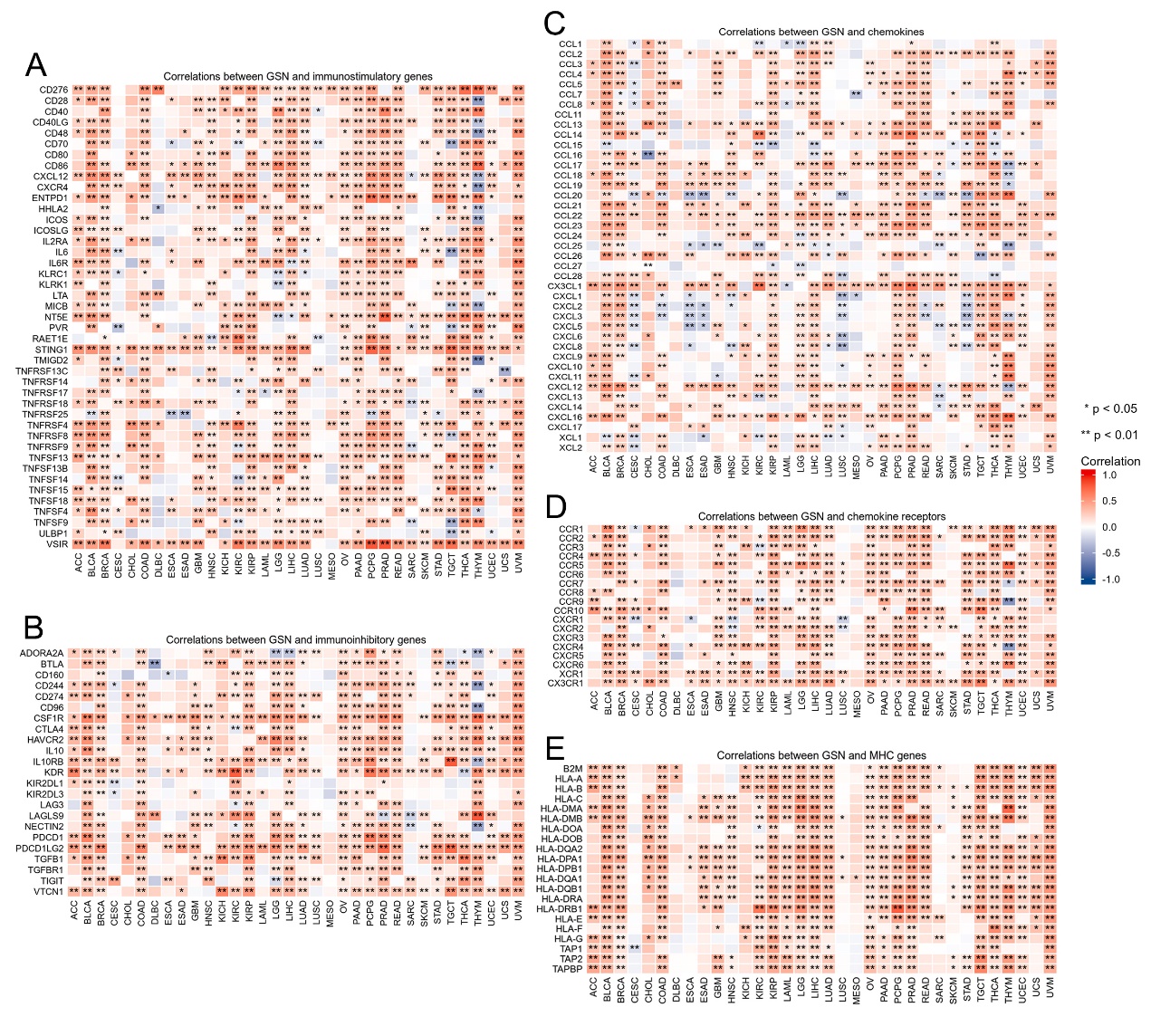


**Supplementary Figure S5.** GSN expression is associated with immune-related genes in various cancers, including immunostimulatory genes **(A)**; immunoinhibitory genes **(B)**; chemokines **(C)**; chemokine receptors **(D)**; MHC genes **(E)**.


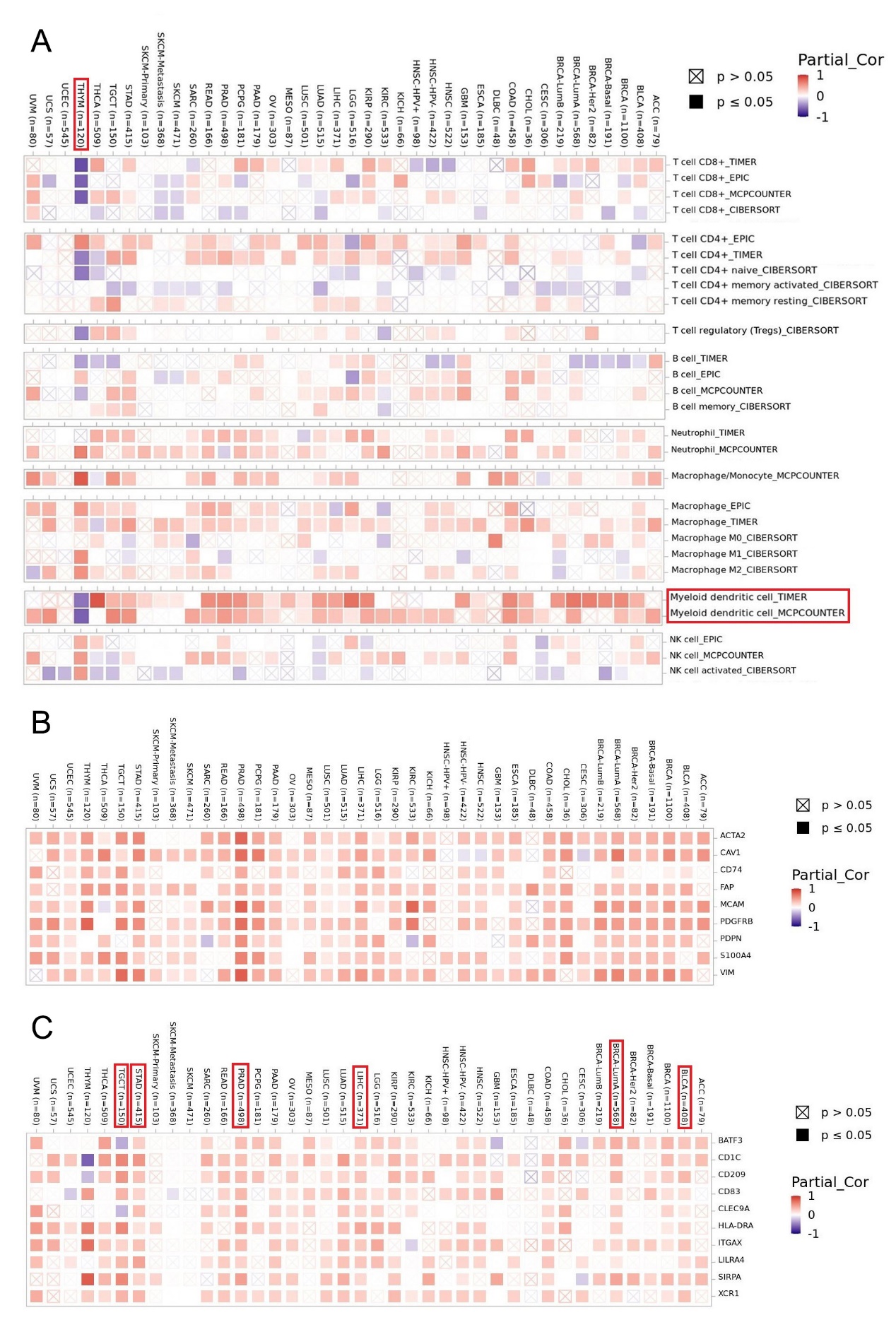
**Supplementary Figure S6.** Associations between immune cell infiltration levels and GSN expression in pan-cancer. **(A)** Correlation analysis of GSN expression with multiple immune cell infiltrates in pan-carcinogenesis, including CD8+ T cells, CD4+ T cells, Tregs, B cells, neutrophils, monocytes, mDCs, macrophages, and NKs. **(B)** Correlation between GSN expression and CAF cell marker expression in pan-carcinoma, including ACTA2, CAV1, CD74, FAP, MCAM, PDGFRB, PDPN, S100A4, and VIM. **(C)** Correlation between GSN expression and mDC cell marker expression in pan-carcinoma, including BATF3, CD1C, CD209, CD83, CLEC9A, HLA-DRA, ITGAX, LILRA4, SIRPA, and XCR1.

**Supplementary Table S1.** Univariate and multivariate Cox analysis of clinical parameters in BLCA **(A)**, CESC **(B)**, KIRC **(C)**, LAML **(D)**, LGG **(E)**, SARC **(F)**, STAD **(G)**, THCA **(H)**.

**Supplementary Table S2.** The top 100 genes associated with GSN.

**Supplementary Table S3.** GO terms and KEGG pathways enriched.
